# Superior Valorisation of *Juglans regia* L. Leaves of Different Maturity through the Isolation of Bioactive Compounds

**DOI:** 10.3390/molecules28217328

**Published:** 2023-10-29

**Authors:** Mihaela Tociu, Fulvia Manolache, Brîndușa Bălănucă, Alina Moroșan, Raluca Stan

**Affiliations:** 1 Department of Organic Chemistry “Costin Neniţescu”, Faculty of Chemical Engineering and Biotechnologies, National University of Science and Technology POLITEHNICA Bucharest, 1–7 Gh. Polizu Street, 011061 Bucharest, Romania; mihaela.tociu@upb.ro (M.T.); brindusa.balanuca@upb.ro (B.B.); alina.morosan@upb.ro (A.M.); 2 National Research and Development Institute for Food Bioresources—IBA Bucharest, 6 Dinu Vintilă Street, 021101 Bucharest, Romania; fulvia.manolache@yahoo.com

**Keywords:** *Juglans regia* L. leaves extracts, total polyphenol content, total antioxidant capacity, bioactive compounds, FT-ICR-MS, waste recovery

## Abstract

Extracts rich in bioactive compounds from natural sources have received great interest due to their great impact on human health. The aim of this research is focused on the obtaining and characterization of several extracts from *Juglans regia* L. leaves in four different maturity phases: young green leaves (YGL), green leaves (GL), mature green leaves (MGL), and yellow leaves (YL), using different solvents: ethanol (e), water (w), or water:ethanol (1:1 (*v*/*v*)—m) by employing several methods: magnetic stirring (MS), ultrasound-assisted (UA), as well as maceration (M). The obtained extracts were quantitatively evaluated through spectrophotometric methods: Total Polyphenol Content (*TPC*-Folin–Ciocalteu assay) and Total Antioxidant Capacity (*TEAC* assay). Phytochemical screening by means of Fourier-Transform Ion–Cyclotron-Resonance High-Resolution Mass Spectrometry (FT-ICR-MS) indicated the presence of 40 compounds belonging to different phytochemical classes: phenolic acids, flavonoids, flavones, flavanones, flavonones, flavanols, vitamins, tereponoid, steroid, anthocyanidin, and other compounds. Based on *TPC* and *TEAC* assays, the water-ethanol mixture was found to be the proper extraction solvent, with the best results being obtained for YL plant material: 146.29 mg GAE/g DM (*TPC*) and 11.67 mM TE/g DM (*TEAC*). This type of extract may be used in various domains, such as the cosmetics industry, the biomedical field, and/or the design of functional foods, relying on their phytochemical composition.

## 1. Introduction

There is a general tendency among the population to improve or maintain their health by using foods and cosmetics containing bioactive compounds of natural origin [1]. Plants can be used in the food industry for their organoleptic and nutritional qualities, as sources of antioxidants to preserve food quality, and also for medicinal purposes, since medicinal herbs are still involved in human healthcare and disease prevention for an important part of the world’s population [2,3].

*Juglans regia* L. belongs to the family Juglandaceae, which includes a few species and is largely spread out around the world [4]. The walnut crops are predominant in moderate-climate regions, being abundant in the North-Western Himalayas of Kashmir, which produces most of the world’s walnuts (around 88% of total walnut production), while the United States, North Africa, western South America, southern Europe, and East Asia are the largest traders of nuts and nut derivatives [4,5].

The *Juglans regia* L. harvests generate agro-forest waste of great value as a source of natural compounds with medicinal properties [6], as the roots [7], bark [8], shoot, leaves, branch, male flowers [9,10], fruits (husk [11,12], septum [13], kernel [14], and kernel skin [15]). Among the identified medicinal properties, we can list antidiabetic, anticancer, antioxidant, antimicrobial, anti-hypersensitivity [16], and UV-protective, anti-inflammatory, and antiaging activities [1]. Each part of the plant leads to extracts with different properties based on their varieties, soil and geographic conditions, and extraction method [17].

Even if it is a by-product or agricultural waste, the walnut leaves contain considerable amounts of phenolic compounds remarkable for their excellent pharmacological and therapeutic properties [18]. They are easily available in large quantities, while the other parts of the tree, such as bark, are not abundant, and plant development depends on them. Walnut leaves serve as a source of phytocompounds and have been extensively used as remedies in folk medicine for the treatment of venous insufficiency, hemorrhoidal symptomatology, anthelmintics, antidiarrheal, depurative, and astringent properties, fungal or microbial infections, and hypoglycemia. Anti-scrofulous, hypotensive, antifungal, keratolytic, hypoglycemic, and sedative activities have also been reported for the extracts derived from walnut leaves [19,20,21,22,23]. In European countries, dried walnut leaves are often used as an infusion, particularly in rural areas. Juglone is the main natural phenolic compound identified in walnut [24,25], being found in fresh walnut leaves [26]. Moreover, the leaves of *Juglans regia* L. are considered a good source of flavonoids [27] and other important bioactive compounds [28,29]. Thus, several studies reported the presence of different valuable compounds in the walnut leaves, such as quercetin 3-o-glucoside and quercetin pentosides, gallic acid, 3-caffeoylquinic acid, 3-*ρ*-coumaroylquinic acid, protocatechuic acid, 4-caffeoylquinic acid, 4-*ρ*-coumaroylquinic acid, *ρ*-coumaric acid quercetin-3-*o*-deoxyhexoside, and other important phenolic derivatives [30,31,32].

Using secondary plant resources to isolate bioactive compounds represents an important way to reduce agricultural waste but also to isolate essential chemicals from biomass. In this regard, the aim of this work was to establish the biological activities of plant extracts obtained from non-conventional sources. Thus, we report a good correlation between the phytochemical compounds of extracts prepared from *Juglans regia* L. leaves in different maturity phases: young green leaves (YGL), green leaves (GL), mature green leaves (MGL), and yellow leaves (YL), identified by FT-ICR-MS and quantified by spectrophotometric methods. Firstly, the optimization of the extraction process was performed (extraction time and method). The optimum conditions were then used for the preparation of different extracts prepared from leaves with different degrees of maturity.

## 2. Results and Discussion

### 2.1. Obtaining and Characterization of Juglans regia *L.* Leaves Extracts

#### 2.1.1. Processing of Plant Material

The plant material was harvested from the same source (*Juglans regia* L. from the Muntenia region, Romania) in different periods of the year. The walnut leaves were processed under the same conditions in order to obtain comparable results. The walnut leaves were dried to a constant mass, and the moisture amount (%wt.) was determined (Table 1). The dried leaves were crushed mechanically and passed through a sieve with a mesh diameter of 1 mm. The obtained products were stored in paper bags at room temperature.

According to the data in Table 1, the moisture content (%wt.) varied greatly (55.17–75.74%) depending on the harvest time, being directly influenced by the atmospheric conditions (temperature and relative humidity). Thus, %wt. decreased by up to 27.16% for MGL, by 14.66% for YL, and by 3.97% for GL compared to YGL. This aspect is very important to estimate the amount of plant material that can result annually, respectively, in raw material to obtain extracts on an industrial scale.

#### 2.1.2. Establishing the Optimal Extraction Parameters

The performance of the extraction method is related to the content of isolated bioactive molecules from the YGL, GL, MGL, and YL (considered initial dry leaves, not. DM) was evaluated considering the total polyphenol content (*TPC*) using the Folin–Ciocalteu method and the Trolox equivalent antioxidant capacity (*TEAC*) using the *TEAC* assay. *TPC* has been expressed in mg gallic acid equivalents (GAE)/g dry matter (DM), and *TEAC* has been expressed in mM Trolox equivalents (TE)/g dry matter (DM).

##### The Influence of the Extraction Time

To identify the optimal extraction time, MGL raw material was first used. This type of plant material was chosen in accordance with Salami et al. [33], who established that the green leaves contain a high amount of phenolic compounds. The plant product (MGL) was extracted with a hydroethanolic mixture by the MS method at different times: 1, 10, 20, 30, 45, and 60 min.

**Figure 1:**
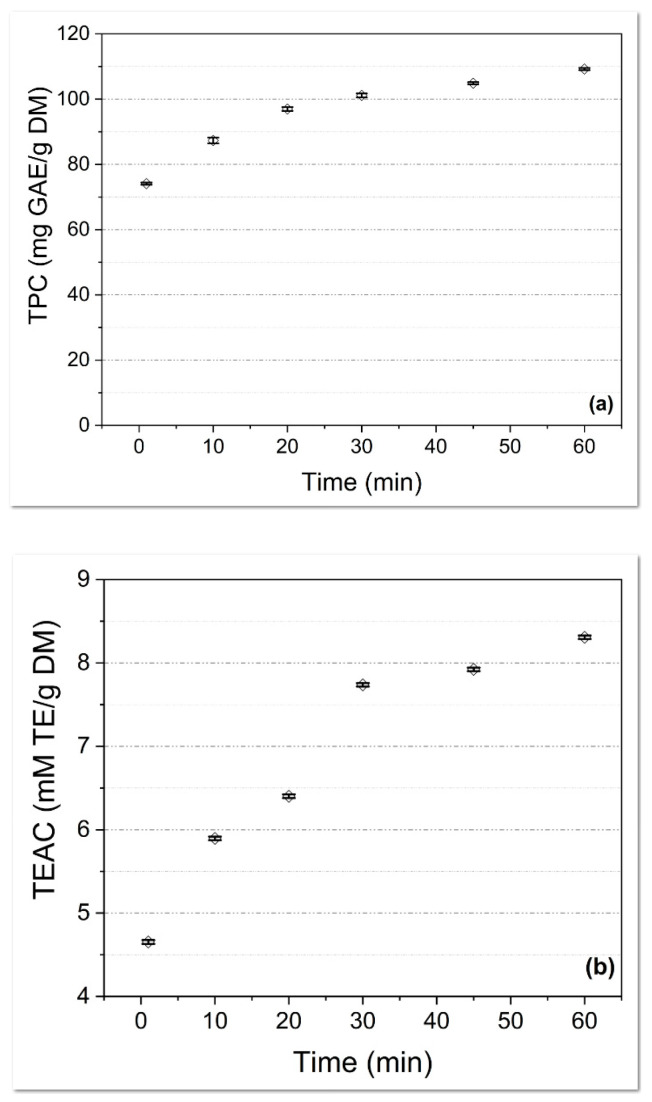
Evaluation of the extraction time for (**a**) *TPC* and (**b**) *TEAC* (MGL extract in hydroethanolic mixture, MS extraction method, different extraction times).

According to reported studies [34], a long extraction time is required to isolate higher amounts of polyphenols. As can be observed, the mean *TPC* values and *TEAC* values increased with the increase in extraction time. The *TPC* values (Figure 1a) ranged from 74.10 ± 0.34 mg GAE/g DM (1 min of extraction) to 109.21 ± 0.34 mg GAE/g DM (60 min of extraction), with a linear increase up to 20 min. The *TEAC* values (Figure 1b) range from 4.65 ± 0.02 mM TE/g DM (after 1 min) to 8.31 ± 0.02 mM TE/g DM (after 60 min), with an almost linear increase up to 30 min. These results indicate the possibility of using a shorter extraction time (less than 60 min) to obtain products with convenient bioactive compound content in the context of a cost-reduced process. This may be argued by the percentage differences between 20 min and, respectively, 60 min of extraction. The *TPC* values for the extract resulted in a 20 min increase of almost 131%, while the *TPC* value for the extract obtained after 60 min increased by almost 147%, with comparisons being made against the *TPC* values for the product obtained after 1 min of extraction. For the *TEAC* results, when the same comparison is made (against the extract resulted from 1 min), the results show an increase of almost 166% for the extract resulted after 30 min and almost 179% for that obtained after 60 min. This small difference of only 13% of *TEAC* values and 16% for *TPC* values suggests the possibility of using shorter extraction times with convenient results in the context of an industrial application.

##### The Influence of the Extraction Solvent and Processing Method

MGL has been subjected to extraction with different solvents: aqueous medium (ultrapure water), organic solvent (absolute ethyl alcohol), and a mixture of them in a ratio of 1:1 (*v*/*v*). The three solvents were used to obtain MGL extracts using all the selected extraction methods: MS (60 min), UA (60 min), and M (24 h).

The extraction solvent greatly influences the type and quantity of polyphenols, being higher in the case of the water-ethanol mixture than for ethanol or water. These results are related to the solubility of the extracted polyphenol species, most of which are probably water-soluble [35]. The *TPC* values (Table 2) range from 27.75 ± 0.59 mg GAE/g DM to 110.39 ± 0.90 mg GAE/g DM. The determined content of total polyphenols was higher compared to previously published reports on the analysis of Juglans regia leaves. In the work by Shah et al., the content of these compounds ranged from 37.61 to 46.47 mg GAE/g (*TPC*) [36], in the work of Untea et al., the *TPC* was 53.94 mg GAE/g [37], and in the work of Jabli et al., it was 103.33 mg GAE/g (*TPC*) [38].

When the experiments were compared regarding the method and solvent used, the highest quantity of polyphenols was obtained using magnetic stirring (60 min) and maceration extraction (24 h) in a water-ethanol solvent mixture. Thus, the influence of the extraction method may be noticed in the context of a more rapid process. The use of water or ethanol as an extraction solvent led to lower phenolic contents for all the extraction methods, contrary to other reported results [39].

Polyphenolic extracts obtained from the MGL raw material were also characterized concerning their ability to interact with free radicals using ABTS assays (Table 2). The radical scavenger activity expressed as Trolox equivalents (TE)/g dry matter (DM) varied between 2.79 ± 0.02 mM TE/g DM and 8.27 ± 0.02 mM TE/g DM. The antioxidant activity of the obtained extracts is similar to that reported by Zurek et al., respectively 9.09 mmol TE/g dry extract (ABTS) [30]. Ethanol and water used for the extractive procedure may promote the extraction of the cyanidins, which have strong antioxidant potential due to their -OH group position within the molecule [40].

Comparing the experimental methods, considering the solvent and time for the extraction of the polyphenolic compounds and antioxidants from the MGL extracts, the obtained results (Table 2) clearly indicate that ultrasound-assisted extraction is more efficient, obtaining the highest value for *TEAC* (8.27 ± 0.02 mM TE/g DM, water/ethanol mixture).

The statistical analysis performed according to a Two-Way ANOVA highlights the importance of the involved method for the polyphenolic content of the obtained extracts. As can be observed from Table 2, both the solvent and the extraction method influence the amount of phytocompounds. Most of the samples resulted from the UA and M methods being statistically different when compared to the reference method, MS. In this context, UA was selected as the extraction method for the extractive experiments, considering the advantages of unconventional processes (ultrasounds, microwaves, and so on), which do not affect the quality of the extracted bioactive compounds. Also, unconventional methods are considered sustainable processes (low energy consumption) [41,42,43].

The mean values of *TPC* for experiments 1–9 presented in Table 2 and corresponding levels of model factors are listed in Table 3. The effects of the considered categorical factors (extraction method and solvent) on total polyphenol content were quantified using a statistical model presented in Equation (1). Predicted values of total polyphenol content (*TPC_pred_*) and related residuals (Δ*TPC*) are also presented in Table 3. The values of the main model characteristic *RMSE* defined by Equation (2) (multiple *R*^2^, adjusted *R*^2^, predicted *R*^2^, adequate precision, *F*, and *p*), encompassed in Table 3, emphasize a good correlation between experimental and predicted *TPC* values. Similar statistics were applied to the antioxidant capacity, and the corresponding statistics are presented in Table 4 using Equations (3) and (4).

In the experiment’s design, categorical factors, extraction method, and solvent were associated with numerical values. To quantify the extraction method for magnetic stirring, A [1] = 1, A [2] = 0, ultrasound-assisted A [1] = 0, A [2] = 1, and maceration, A [1] = −1, A [2] = −1. To quantify the solvent, ethanol is denoted B [1] = 1, B [2] = 0; water is B [1] = 0, B [2] = 1; water-ethanol: B [1] = −1, B [2] = −1.

(1) $${\mathrm{TPC}}_{\mathrm{pred}}=76.27-5.26\cdot A\left[1\right]-0.3945\cdot A\left[2\right]-40.11\cdot B\left[1\right]+7.63\cdot B\left[2\right]$$

(2) $$RMSE=\sqrt{\frac{\overset{N}{\underset{i=1}{\sum }}\Delta TPC_{i}^{2}}{N}}=\sqrt{\frac{\overset{9}{\underset{i=1}{\sum }}{\left(TPC_{i}-{\mathrm{TPC}}_{{\mathrm{pred}}_{i}}\right)}^{2}}{9}}$$

(3) $$RMSE=\sqrt{\frac{\overset{N}{\underset{i=1}{\sum }}\Delta TEAC_{i}^{2}}{N}}=\sqrt{\frac{\overset{9}{\underset{i=1}{\sum }}{\left(TEAC_{i}-{\mathrm{TEAC}}_{{\mathrm{pred}}_{i}}\right)}^{2}}{9}}$$

(4) $$TEAC_{pred}=5.35-0.0126\cdot A\left[1\right]+0.4061\cdot A\left[2\right]-2.29\cdot B\left[1\right]+0.11\cdot B\left[2\right]$$

After performing a numerical optimization of the proposed model and imposing a maximized value of both *TPC* and *TEAC*, the best extraction process was considered ultrasound-assisted in hydroethanolic solvent with a good desirability value of 0.957.

##### The Obtaining of Walnut Leaf Extracts (YGL, GL, MGL, YL)—The Influence of the Extraction Solvent

For this experimental phase, the plant material is represented by walnut leaves in different stages of maturity, from young green leaves to yellow leaves, which are harvested at different times of the year (according to Table 1). The studied walnut leaves (YGL, GL, MGL, and YL) were subjected to extraction with the same three solvents: water, ethanol, and their mixture, respectively (1:1, *v*/*v*).

All 12 experiments were performed using ultrasound-assisted extraction (UA) for 60 min.

*TPC* values range from 0.73 ± 0.34 to 146.29 ± 0.90 mg GAE/g DM, depending on the studied extract. *TEAC* values range from 0.03 ± 0.00 to 11.67 ± 0.02 mM TE/g DM. The lowest values for *TPC* and *TEAC* were recorded for green leaf extract (GL) in ethanol, and the highest values were recorded for yellow leaf extract (YL) in water:ethanol. In an unexpected manner, these results indicate that YL-type plant material contains high amounts of biologic compounds (phenolic species) with great antioxidant activity.

Beside these maxim values registered for the YL type material, MGL also indicated a great content of polyphenol species and great antioxidant capacity, irrespective of the used extraction solvent. Moreover, in a sustainable and economical context related to industrial applications, water demonstrates to be a good choice to isolate polyphenols from MGL and YL as well. However, reported performant polyphenol water extractions of different algae required more complex extraction procedures, such as pressurized liquid extraction assisted by pulsed electric fields [44,45].

Statistical analysis (Two-Way ANOVA) on *TPC* values considering both leaves maturity and the solvent used shows that both factors influenced the total polyphenol amount, with similarities being observed for extract prepared in ethanol and water or water and hydroethanolic mixture, or from MGL and YL or YGL and GL raw material. In the case of *TEAC* values, statistical analysis showed that the maturity of the leaves influences the antioxidant activity, with similarities in the cases of MGL and YL or YGL and GL, while the solvent does not significantly influence *TEAC* values.

The mean values of *TPC* for experiments 1–12 are presented in Table 5, and the corresponding levels of model factors are listed in Table 6. The effects of considered categorical factors (leaves maturity and solvent) on total polyphenol content were quantified using a statistical model presented in Equation (5). Predicted values of total polyphenol content (*TPC_pred_*) and related residuals (Δ*TPC*) are also presented in Table 6. The values of the main model characteristic *RMSE* defined by Equation (6), multiple *R*^2^, adjusted *R*^2^, predicted *R*^2^, adequate precision, *F*, and *p*), encompassed in Table 6, emphasize a good correlation of experimental and predicted *TPC* values. Similar statistics were applied to the antioxidant capacity, and the corresponding statistics are presented in Table 7 using Equations (7) and (8).

In the experiment’s design, categorical factors such as leaf maturity and solvent were associated with numerical values. To quantify leaf maturity: YGL (A [1] = 1, A [2] = 0; A [3] = 0), GL (A [1] = 0, A [2] = 1; A [3] = 0), MGL (A [1] = 0, A [2] = 0; A [3] = 1), and YL (−1, −1, −1). To quantify the solvent, ethanol is denoted B [1] = 1, B [2] = 0; water is B [1] = 0, B [2] = 1; water−ethanol: B [1] = −1, B [2] = −1.

(5) $$\sqrt{{\mathrm{TPC}}_{\mathrm{pred}}}=5.83-3.45\cdot A\left[1\right]-3.59\cdot A\left[2\right]+2.71\cdot A\left[3\right]-1.83\cdot B\left[1\right]-0.0408\cdot B\left[2\right]$$

(6) $$RMSE=\sqrt{\frac{\overset{N}{\underset{i=1}{\sum }}\Delta TPC_{i}^{2}}{N}}=\sqrt{\frac{\overset{12}{\underset{i=1}{\sum }}{\left(TPC_{i}-{\mathrm{TPC}}_{{\mathrm{pred}}_{i}}\right)}^{2}}{12}}$$

(7) $$RMSE=\sqrt{\frac{\overset{N}{\underset{i=1}{\sum }}\Delta TEAC_{i}^{2}}{N}}=\sqrt{\frac{\overset{12}{\underset{i=1}{\sum }}{\left(TEAC_{i}-{\mathrm{TEAC}}_{{\mathrm{pred}}_{i}}\right)}^{2}}{12}}$$

(8) $$\log\left({\mathrm{TEAC}}_{\mathrm{pred}}\right)=-0.2793-1.05\cdot A\left[1\right]-1.12A\left[2\right]+1.01\cdot A\left[3\right]-0.2025\cdot B\left[1\right]-0.0426\cdot B\left[2\right]$$

A numerical optimization of the proposed model was performed, imposing a maximized value of both *TPC* and *TEAC*. The best extraction factors were yellow leaves (YL) and hydroethanolic solvent, with an excellent desirability value of 0.998.

### 2.2. Phytochemical Characteristics of Walnut Leaves Extracts

#### Identification of Phytochemical Compounds from Walnut Leaf Extracts

Following FT-ICR-MS analysis (ESI+, according to Figures S4_1–S4_23 in Supplementary Materials), we have identified for the studied YGL, GL, MGL, and YL extracts a total number of 40 different compounds based on their molecular formulas, the identification being made based on their specific molecular weight (Table 8). These results were obtained in a relatively short time by direct analysis of the sample without prior processing.

All the obtained and earlier discussed MGL extracts were subjected to the FT-ICR-MS analysis, both those obtained by magnetic stirring (MS, 1–60 min of extraction, water:ethanol mixture as extraction solvent, or MS, 60 min of extraction in water or ethanol) or by using ultrasound treatment (UA, 60 min) or maceration (M, 24 h).

Also, YGL, GL, and YL extracts obtained through UA treatment (60 min) using the three extraction solvents (water, ethanol, or their mixture) were evaluated through the FT-ICR-MS analysis. The obtained results are summarized in Table 8, where the present compounds are marked with the “+” sign, while the compounds that were not identified in the studied extracts are marked with the “−” sign. In Table 8, the analyzed samples were numbered from 1 to 23.

As can be observed from the experimental results from Table 8, there are no major compositional differences for 1–6 extract samples, representing the different MGL products obtained through the MS method in hydroalcoholic solvent by varying the extraction time (1–60 min). Also, by comparing the compositions of the hydroalcoholic MGL extracts (MGL, m) obtained by different extraction methods (MS, samples 7, 9, 12; UA, samples 8, 10, 13; M, samples 6, 11, 14), there were no notable differences. Thus, assume that both the extraction time and the extraction method do not influence the composition of the resulting extracts. However, the same similarity is no longer preserved for aqueous and ethanolic extracts; these results are explained by the different solubility of the phytochemical compounds.

FT-ICR-MS results indicate the presence of common compounds in all extracts (samples 1–23, according to Table 8): chlorogenic acid, 3-p-coumaroylquinic acid, quercetin 3-o-arabinoside, quercetin 3-rhamnoside, quercetin, epigallocatechin gallate kaempferol, kaempferol 3-o-arabinoside, kaempferol-3-o-rhamnoside, catechin hydrate, luteolin, catechin, taxifolin, and specific compounds also for some extract samples: ellaigic acid, cyanidin-3-glucoside chloride, resveratrol, azelaic acid. Related to the solvent used for the extraction, most phytochemicals were identified in both ethanolic and aqueous samples. However, some compounds were not identified for the ethanolic ones: caffeic acid, ferulic acid, juglone, myricetin, isokaempferide, hyperoside, fisetinidol, guibourtinidol, pelargonidin, and quinic acid, the explanation being their insolubility in this organic solvent [46].

When comparing the composition of the extracts obtained starting from walnut leaves with different degrees of maturity (UA extraction, 60 min), FT-ICR-MS analysis reveals the lack of certain phytochemical compounds for the YGL samples (irrespective of the used extraction solvent), such as pelargodin, p-coumaric acid, sinapate, hesperetin, ferulic acid, fisetinidol, and guibourtinidol. But it is important to mention the presence of azelaic acid in the ethanolic YGL extract. Also, another important feature of some walnut leaf extracts is the presence of sinapate, p-coumaric acid, epigallocatechin, ferulic acid, and fisetinidol, which were identified exclusively in mature leaves, MGL and YL, respectively. This behavior suggests that the appearance of these bioactive compounds in leaves is associated with the processes of maturation and aging.

Following FT-ICR-MS analysis (ESI+), the results show a greater diversity in the phytochemical profile of the MGL samples; the color changes from green to yellow (due to the aging process of the leaves), leading to changes in composition and the appearance of new compounds with biologically active value. However, by correlating all the experimental information, YL extracts indicate high antioxidant capacity, which denotes the presence of large or important quantities of phenolic species of great interest and antioxidant activity. The composition of the phytochemical compounds in the natural extracts is very important for the selection of further applications. Related to the studied walnut leaf extracts, due to their compositional diversity, they are recommended to be used in food and non-food products. The so varied composition of phytochemicals belonging to different classes of compounds was clearly suggested from the first experimental phase by the different colors of the various obtained extracts, as can be seen from the Supplementary Material—Figure S1.

The phytochemical compounds identified in the studied walnut leaf extracts have a series of biological activities. These were established and published in several studies, listed in the Supplementary Material (Table S1) [6,34,39,47,48,49,50,51,52,53,54,55,56,57,58,59,60]. The main compounds identified within this study belong to the following phytochemical classes: phenolic acids, flavonoids, flavones, flavanones, flavonones, flavanols, vitamins, tereponoids, steroids, anthocyanidins, and other compounds.

By corroborating the experimental data obtained within this study with other literature reports, we can state that walnut leaves contain a series of compounds with important biological activity, irrespective of the degree of the leaf’s maturity.

In this regard, these plant materials, considered to be residue/byproduct are strongly recommended to be more investigated and directed to different applications in the food or non-food industries as sources of polyphenolic and antioxidant species.

## 3. Materials and Methods

### 3.1. Plant Material

Walnut leaves (*Juglans regia* L.) were harvested between October 2022 and June 2023 from the Muntenia region (Romania—latitude: 44,44869 N 44°26′55″; longitude: 26,0791 E 26°4′44″), an area with a moderate-continental climate with an average annual temperature of 10–11 °C. So that there are no changes due to the source and the soil, the leaves were harvested from the same tree, a 7-year-old walnut, located in a place protected from direct sunlight, with harvesting being carried out at different times of leaf maturity. The fresh leaves were cleaned to remove dust and impurities, then they were dried at room temperature (T = 25 °C) in a place protected from direct sunlight until they reached a constant mass for 7–10 days. Size reduction was achieved using a mechanical grinder. Then, the dried plant material was sieved through a 1 mm mesh sieve. The dried and ground leaves were stored in paper bags in a dry place until use.

### 3.2. Chemicals

Ethanol (96% purity, Sigma Aldrich, St. Louis, MO, USA) was used as received; ultrapure water was also used for the extraction experiments. Approximately 2,2′-Azino-bis (3-ethylbenzo-thiazoline-6-sulphonic acid) (ABTS), 6-hydroxy-2,5,7,8-tetramethylchroman-2-carboxylic acid (Trolox), gallic acid (GA), sodium carbonate, and potassium persulfate were purchased from Sigma-Aldrich. Folin–Ciocalteu reagent was purchased from Merck. These chemicals were analytical grade, and the water was Milli-Q, with a resistivity of 18.2 MΩ·cm at 25 °C.

### 3.3. Preparation of Plant Extracts

Plant extracts were obtained starting from walnut leaves in four different phases of maturity: young green leaves (YGL), green leaves (GL), mature green leaves (MGL), and yellow leaves (YL). The extraction of biological compounds from the four plant materials was performed by varying the extraction method: magnetic stirring (MS), ultrasound-assisted extraction (UA), as well as maceration (M). Different extraction parameters were tested. Thus, as extraction media, ultrapure water (w), ethanol (e), or a water-ethanol mixture (1:1, *v*/*v*, m) were employed. Several extraction times were also tested: 1, 10, 20, 30, 45, 60 min when MS or UA have been involved (1000 rpm, MSH 300, BIOSAN for MS; Elmasonic S10 Elma ultrasound bath, power level of 30 W for UA) and 24 h for maceration (in the dark, room temperature). The ratio of walnut leaves (YGL, GL, MGL, and YL) to solvent was 1:200 (g/mL). After leaf processing, the obtained mixtures were filtered using medium-porosity filter paper and then centrifuged at 4000 rpm (Nahita centrifuge model 2640/12) for 10 min. The supernatant was collected and stored at 4 °C until the analysis.

### 3.4. Determination of Total Polyphenol Content (TPC) Using Spectrophotometric Method

The determination of total polyphenol content (*TPC*) was performed based on the Folin–Ciocalteu (FC) reaction, according to ISO 14502-1:2005(E) [61]. The studied extracts (0.01 mL, obtained from YGL, GL, MGL, and YL) were diluted with ultrapure water (0.99 mL), then 5 mL of Folin–Ciocalteu reagent (10%) was added, and after 4 min, 4 mL of Na_2_CO_3_ solution (7.5%) was added. The mixtures were incubated for 1 h at room temperature in a dark place.

The absorbance of each sample was recorded at λ = 765 nm using a Helios Beta UV–Vis spectrophotometer (Thermo Electron Corporation, Waltham, MA, United States) with Thermo Scientific™ VISION pro™ software. The analysis of each extract was performed in triplicate.

The results were expressed as mg of gallic acid equivalents (GAE) per g of dry matter (mg GAE/g DM) using a standard curve corresponding to a gallic acid solution of 0.5–50 mg/mL, with *R*^2^ = 0.9986 (y = 0.0168x + 0.0141) (the standard curve is presented as Supplementary Material—Figure S2).

### 3.5. Evaluation of Trolox Equivalent Antioxidant Capacity (TEAC) Using the Spectrophotometric Method

The turquoise-colored ABTS radical (ABTS^•+^) results from the reaction of a strong oxidizing agent (potassium persulfate) with the ammonium salt of 2,2′-azino-bis(3-ethylbenzothiazoline 6-sulfonic acid). Under the reaction of the antioxidant, the color intensity is reduced to colorless [62]. The concentration of the reagents in aqueous solutions was 7 mM for ABTS and 2.45 mM for potassium persulfate. After 16 h (kept in the dark at room temperature), the reaction mixture was normalized by adjusting the absorbance value to 0.68 (± 0.02) at 734 nm with ethanol. In a 1 cm cuvette, 990 µL ABTS^•+^ solution and 10 µL standard/extract were homogenized, and the absorbance was read after 1 min. For the calibration curve, solutions of Trolox in ethanol at concentrations between 0.25 and 1.25 mM were prepared. The equation of the linear domain of the curve for inhibition percentage vs. Trolox concentration was: y = 63.614x + 1.7268 (where y = percentage of inhibition and x = concentration of standard) and *R*^2^ = 0.9992 (the calibration curve is presented as Supplementary Material—Figure S3). For the spectral measurements, a UV–Vis Spectrophotometer UVmini-1240 instrument from SHIMADZU (Kyoto, Japan) was used. The inhibition of the ABTS^•+^ radical was calculated as follows:

%I ABTS^•+^ = [(OD_i_ − OD_f_)/OD_i_] × 100

where OD_i_ represents the absorbance of the reference solution (990 μL ABTS^•+^ normalized solution) and OD_f_ is the absorbance of the tested samples (990 μL ABTS^•+^ normalized solution, 10 μL standard/extract). All measurements were performed in triplicate, and the antioxidant activity was expressed as mmoli/g DM Trolox equivalents.

### 3.6. Fourier-Transform Ion-Cyclotron-Resonance High-Resolution Mass Spectrometry (FT-ICR-MS Method)

Analysis was performed with the Fourier-Transform Ion-Cyclotron-Resonance High-Resolution Mass Spectrometer (FT-ICR-MS) system type SolariX-XR QqqFT-ICR HR (Bruker Daltronics, Bremen, Germany) with a 15T superconducting magnet for both negative and positive ionization [63]. Each sample was introduced by direct infusion and positive ESI ionization, with a sample flow rate of 120 µL/h, a nebulization gas pressure (N_2_) of 2.2 bar at 180 °C, and a flow rate of 4 L/min. The spectra were recorded over a mass range between 46 and 1200 amu at a source voltage of 5700 V.

The samples were prepared by dissolving the extracts in solvents: ultrapure water or methanol or ultrapure water:methanol 1:1 (*v*/*v*) (Sigma-Aldrich, Taufkirchen, Germany), depending on the solvent used for extraction. A 100 µL sample was taken, adding 10 µL of formic acid and diluting to 10 mL. The sample thus obtained was injected into the system. Based on the molecular formula, ESI+ (1M + nH) ionization templates were generated (Bruker Compass Data Analysis) and identified in the obtained spectrograms.

### 3.7. Statistical Analysis

Statistical analysis was performed using a Two-Way ANOVA followed by a post-hoc Tukey Test from Origin Lab 2021 software (Origin Lab Corporation, Northampton, MA, USA), and differences were considered significant at *p* < 0.05.

Statistical models employed to determine effect factors on *TPC* and *TEAC* were determined using Design Expert 13 software (StatEase, Minneapolis, MN, USA).

## 4. Conclusions

The experimental results obtained in the current study showed that the investigated walnut leaves, with different degrees of maturity (young green leaves—YGL; green leaves—GL; mature green leaves—MGL; yellow leaves—YL), contain significant amounts of polyphenolic compounds, especially YL and MGL, irrespective of the extraction solvent used.

The best results were obtained for the hydroethanolic YL extract obtained by ultrasound-assisted extraction for 60 min. The YL extraction product registered a *TPC* of 146.29 ± 0.90 mg GAE/g DM and a *TEAC* of 11.67 ± 0.02 mM TE/g DM. Thus, the starting material, yellow leaves, considered a real waste from walnut crops, proves to be a valuable material for the isolation of bioactive compounds.

All the studied extracts were characterized by means of FT-ICR-MS, establishing their phytochemical profiles and identifying a total of 40 different compounds. It was found that their composition is considerably influenced by the solvent used, and the different plant materials involved in the extractive processes contain different biological molecules. Also, it was noticed that the number and diversity of the phytochemicals increased over time. YL extracts (from the more mature leaf type) indicated a varied profile of phenolic compounds and contained the highest total flavonoid content, which explains the greater antioxidant activity registered for these samples.

Statistical analysis (Two-Way ANOVA) indicates that the extraction solvent and the leaf’s maturity degree influence the total polyphenol content and the antioxidant capacity of each extract.

The current research provides a background for further studies and, respectively, the utilization of the different walnut leaf extracts based on their phytochemical profiles, being adaptable for the food industry (e.g., treatment and improvement of various aliments) or non-food applications (as nutraceuticals).

This research study dealt with notable aspects regarding the obtaining of the walnut leaves’ extracts and their general composition through qualitative determinations. Perspective works on more detailed analysis related to the antioxidant capacity, quantitative evaluation of the isolated phytochemical compounds, and their possible applications are envisaged.

## Figures and Tables

**Table 1:** The percentage of moisture in the samples varies according to the degree of maturity.

Vegetable Material	Harvest Date	Moisture Content, %
young green leaves (YGL)	15 May 2023	75.74 ± 0.03
green leaves (GL)	26 June 2023	72.73 ± 0.02
mature green leaves (MGL)	14 October 2022	55.17 ± 0.04
yellow leaves (YL)	15 November 2022	64.64 ± 0.03

**Table 2:** *TPC* and *TEAC* of MGL extracts (different solvents and methods).

Method	Solvent	*TPC* *	*TEAC* **
		mg GAE/g DM	mM TE/g DM
magnetic stirring (MS)	ethanol	27.76 ± 0.59 a	2.88 ± 0.02 a
	water	76.08 ± 0.90 b	5.60 ± 0.02 b
	water:ethanol	109.21 ± 0.34 c	7.53 ± 0.02 c
ultrasounds assisted extraction (UA)	ethanol	38.40 ± 0.59 a	3.49 ± 0.02 a
	water	82.58 ± 0.90 b	5.49 ± 0.02 b
	water:ethanol	106.65 ± 0.34 c	8.27 ± 0.02 c
maceration (M)	ethanol	42.35 ± 0.90 a	2.79 ± 0.02 a
	water	93.04 ± 0.34 b	5.28 ± 0.02 b
	water:ethanol	110.39 ± 0.90 c	6.79 ± 0.02 c

* *TPC*—Total polyphenol content; ** *TEAC*—Trolox equivalent antioxidant capacity; DM—dry matter; GAE—gallic acid equivalents; TE—Trolox equivalents; different letters (a–c) show significantly different samples according to Two-Way ANOVA (*p* < 0.05) followed by a post-hoc Tukey Test.

**Table 3:** Experimental and predicted values of total polyphenol content at different levels of extraction process factors and relevant statistics correspond to the regression model.

No. Exp.	Extraction Method	Solvent	*TPC* (mg GAE/g DM)	*TPC_pred_* (mg GAE/g DM)	Δ*TPC* (mg GAE/g DM)
1	MS	ethanol	27.75	30.91	−3.16
2	MS	water	76.08	78.64	−2.56
3	MS	water:ethanol	109.21	103.49	5.72
4	UA	ethanol	38.40	35.77	2.63
5	UA	water	82.58	83.50	−0.92
6	UA	water:ethanol	106.65	108.36	−1.71
7	M	ethanol	42.35	41.82	0.53
8	M	water	93.04	89.55	3.48
9	M	water:ethanol	110.39	114.40	−4.01
				*RMSE*	3.134
				*R* ^2^	0.990
				Adjusted *R*^2^	0.979
				Predicted *R*^2^	0.950
				Adequate Precision	23.82
				*F*	94.34
				*p*	0.0003

*TPC*—mean values of three replicates for total polyphenol content; *TPC_pred_*_—_predicted *TPC* values computed from Equation (1); Δ*TPC*—residual; *RMSE*—root mean square error determined using Equation (2); *R*^2^—coefficient of multiple determination.

**Table 4:** Experimental and predicted values of antioxidant activity at different levels of extraction process factors and relevant statistics correspond to the regression model.

No. Exp.	Extraction Method	Solvent	*TEAC* (mg TE/g DM)	*TEAC_pred_* (mg TE/g DM)	Δ*TEAC* (mg TE/g DM)
1	MS	ethanol	2.88	3.04	−0.1628
2	MS	water	5.60	5.44	0.1526
3	MS	water:ethanol	7.53	7.52	0.0102
4	UA	ethanol	3.49	3.46	0.0315
5	UA	water	5.49	5.86	−0.3687
6	UA	water:ethanol	8.27	7.93	0.3373
7	M	ethanol	2.79	2.66	0.1313
8	M	water	5.28	5.06	0.2162
9	M	water:ethanol	6.79	7.13	−0.3474
				*RMSE*	0.232
				*R* ^2^	0.985
				Adjusted *R*^2^	0.969
				Predicted *R*^2^	0.922
				Adequate Precision	20.30
				*F*	63.89
				*p*	0.0007

*TEAC*—mean values of three replicates for total polyphenols content; *TEAC_pred_*—predicted *TPC* values computed from Equation (3); Δ*TEAC*—residual; *RMSE*—root mean square error determined using Equation (4); *R*^2^—coefficient of multiple determination.

**Table 5:** *TPC* and *TEAC* of YGL, GL, MGL, YL extracts using different solvents (UA, 60 min).

Type of Leaves	Solvent	*TPC* *	*TEAC* **
		mg GAE/g DM	mM TE/g DM
YGL	ethanol	1.32 ± 0.34 a	0.03 ± 0.00 a
	water	2.90 ± 0.59 ab	0.04 ± 0.00 a
	water:ethanol	18.28 ± 0.59 b	0.09 ± 0.00 a
GL	ethanol	0.73 ± 0.34 a	0.03 ± 0.00 a
	water	3.49 ± 0.59 ab	0.03 ± 0.00 a
	water:ethanol	16.90 ± 0.90 b	0.08 ± 0.00 a
MGL	ethanol	38.40 ± 0.59 c	3.50 ± 0.02 b
	water	82.58 ± 0.90 cd	5.50 ± 0.02 b
	water:ethanol	106.65 ± 0.34 c	8.27 ± 0.02 b
YL	ethanol	62.66 ± 0.59 c	4.74 ± 0.02 b
	water	110.20 ± 0.34 cd	7.76 ± 0.02 b
	water:ethanol	146.29 ± 0.90 c	11.67 ± 0.02 b

* *TPC*—Total polyphenol content; ** *TEAC*—Trolox equivalent antioxidant capacity; DM—dry matter; GAE—gallic acid equivalents; TE—Trolox equivalents; different letters (a–d) show significantly different samples according to Two-Way ANOVA (*p* < 0.05) followed by a post-hoc Tukey Test.

**Table 6:** Experimental and predicted values of total polyphenols content at different levels of extraction process factors and relevant statistics correspond to the regression model.

No. Exp.	Plant Maturity	Solvent	*TPC^0.5^* (mg GAE/g DM)	*TPC_pred_^0.5^* (mg GAE/g DM)	Δ*TPC*^0.5^ (mg GAE/g DM)
1	YGL	ethanol	12.10	12.04	0.0531
2	YGL	water	1.70	2.34	−0.6326
3	YGL	water:ethanol	9.09	8.50	0.5912
4	GL	ethanol	4.28	4.25	0.0273
5	GL	water	7.92	8.34	−0.4218
6	GL	water:ethanol	0.7297	0.4047	0.3250
7	MGL	ethanol	4.11	4.11	0.0023
8	MGL	water	1.15	0.5443	0.6052
9	MGL	water:ethanol	6.20	6.71	−0.5084
10	YL	ethanol	10.33	10.41	−0.0827
11	YL	water	10.50	10.13	0.3687
12	YL	water:ethanol	1.87	2.20	−0.3273
				*RMSE*	0.398
				*R* ^2^	0.9895
				Adjusted *R*^2^	0.9808
				Predicted *R*^2^	0.9581
				Adequate Precision	29.15
				*F*	113.29
				*p*	<0.0001

*TPC*—mean values of three replicates for total polyphenol content; *TPC_pred_*—predicted *TPC* values computed from Equation (5); Δ*TPC*—residual; *RMSE*—root mean square error determined using Equation (6); *R*^2^—coefficient of multiple determination.

**Table 7:** Experimental and predicted values of antioxidant activity at different levels of extraction process factors and relevant statistics correspond to the regression model.

No. Exp.	Plant Maturity	Solvent	log(*TEAC*) (mg TE/g DM)	log(*TEAC_pred_*) (mg TE/g DM)	log(Δ*TEAC*) (mg TE/g DM)
1	YGL	ethanol	1.07	1.12	−0.0556
2	YGL	water	−1.39	−1.37	−0.0168
3	YGL	water:ethanol	0.7399	0.6910	0.0489
4	GL	ethanol	−1.05	−1.08	0.0351
5	GL	water	0.6760	0.6750	0.0010
6	GL	water:ethanol	−1.60	−1.60	0.0051
7	MGL	ethanol	−1.07	−1.15	0.0817
8	MGL	water	−1.55	−1.53	−0.0183
9	MGL	water:ethanol	0.5433	0.5311	0.0122
10	YL	ethanol	0.9176	0.9787	−0.0612
11	YL	water	0.8896	0.8350	0.0546
12	YL	water:ethanol	−1.53	−1.44	−0.0867
				*RMSE*	0.049
				*R* ^2^	0.998
				Adjusted *R*^2^	0.966
				Predicted *R*^2^	0.922
				Adequate Precision	55.98
				*F*	615.14
				*p*	<0.0001

*TEAC*—mean values of three replicates for total polyphenol content; *TEAC_pred_*—predicted *TPC* values computed from Equation (7); Δ*TEAC*—residual; *RMSE*—root mean square error determined using Equation (8); *R*^2^—coefficient of multiple determination.

**Table 8:** The phytochemical profiles of YGL, GL, MGL, and YL extracts resulted from the FT-ICR-MS method.

Compound	Theoretical *m*/*z* (ESI+)	Type of Extract																										
		MGL *															YGL **			GL ***			MGL *			YL ****		
		Extraction Method																										
		MS (m)						MS			UA			M			UA											
		Extraction Time (min)						Extraction Solvent (60 min)									Extraction Solvent (60 min)											
		1	10	20	30	45	60	e	w	m	e	w	m	e	w	m	e	w	m	e	w	m	e	w	m	e	w	m
		Sample Number																										
		1	2	3	4	5	6	7	8	6	9	10	11	12	13	14	15	16	17	18	19	20	9	10	11	21	22	23
Caffeic acid	181.049535	−	+	+	+	+	+	−	+	+	−	+	+	−	+	+	−	−	+	−	−	+	−	+	+	−	+	+
Caftaric acid+Na	335.037353	+	+	+	+	+	+	−	+	+	−	+	+	−	+	+	−	+	−	−	+	−	−	+	+	−	+	−
Chlorogenic acid	355.102359	+	+	+	+	+	+	+	+	+	+	+	+	+	+	+	+	+	+	+	+	+	+	+	+	+	+	+
Ellagic acid	303.013544	−	−	−	−	−	−	−	−	−	−	−	−	−	−	−	−	+	−	−	+	−	−	−	−	−	−	−
Ferulic acid	196.065185	+	+	+	+	+	+	−	+	+	−	+	+	−	+	+	−	−	−	−	+	−	−	+	+	−	+	+
Juglone	175.038971	+	+	+	+	+	+	−	+	+	−	+	+	−	+	+	−	−	+	−	−	+	−	+	+	−	+	+
p-coumaric acid	165.054621	+	+	+	+	+	+	−	+	+	−	+	+	−	+	+	−	−	−	−	−	−	−	+	+	−	+	+
Sinapate	225.075750	+	+	+	+	+	+	−	+	+	−	+	+	−	+	+	−	−	−	−	−	−	−	+	+	−	+	+
3-p-coumaroylquinic acid	339.107444	+	+	+	+	+	+	+	+	+	+	+	+	+	+	+	+	+	+	+	+	+	+	+	+	+	+	+
Quercetin 3-o-arabinoside	435.092188	+	+	+	+	+	+	+	+	+	+	+	+	+	+	+	+	+	+	+	+	+	+	+	+	+	+	+
Quercetin 3-rhamnoside	449.107838	+	+	+	+	+	+	−	+	+	+	+	+	+	+	+	+	+	+	+	+	+	+	+	+	+	+	+
Quercetin 3-o-pentoside	567.134447	−	−	−	−	−	−	−	+	−	−	+	−	−	−	−	−	−	−	−	−	−	−	+	−	−	+	−
Quercetin	303.049929	+	+	+	+	+	+	+	+	+	+	+	+	+	+	+	+	+	+	+	+	+	+	+	+	+	+	+
Myricetin	319.044844	+	+	+	+	+	+	−	+	+	−	+	+	−	+	+	−	−	+	−	−	+	−	+	+	−	+	+
Kaempferol	287.055014	+	+	+	+	+	+	+	+	+	+	+	+	+	+	+	+	+	+	+	+	+	+	+	+	+	+	+
Kaempferol 3-o-arabinoside	419.097273	+	+	+	+	+	+	+	+	+	+	+	+	+	+	+	+	+	+	+	+	+	+	+	+	+	+	+
Kaempferol-3-o-rhamnoside	433.112923	+	+	+	+	+	+	+	+	+	+	+	+	+	+	+	+	+	+	+	+	+	+	+	+	+	+	+
Isokaempferide	301.070665	+	+	+	+	+	+	−	+	+	−	+	+	−	+	+	−	−	+	−	+	+	−	+	+	−	+	+
Catechin hydrate	309.096879	+	+	+	+	+	+	+	+	+	+	+	+	+	+	+	+	+	+	+	+	+	+	+	+	+	+	+
Hyperoside	465.102753	+	+	+	+	+	+	−	+	+	−	+	+	−	+	+	+	+	−	−	+	+	−	+	+	−	+	+
Luteolin	287.055014	+	+	+	+	+	+	+	+	+	+	+	+	+	+	+	+	+	+	+	+	+	+	+	+	+	+	+
Naringenin	273.075750	+	+	+	+	+	+	+	+	+	+	+	+	+	+	+	−	−	+	−	+	+	+	+	+	+	+	+
Hesperetin	303.086812	+	+	+	+	+	+	+	+	+	+	+	+	+	+	+	−	−	+	−	−	+	+	+	+	−	+	+
Catechin	291.086315	+	+	+	+	+	+	+	+	+	+	+	+	+	+	+	+	+	+	+	+	+	+	+	+	+	+	+
Epigallocatechin	307.081229	−	−	+	+	+	+	−	+	+	−	+	+	−	+	+	−	−	−	−	−	−	−	+	+	+	+	+
Epigallocatechin gallate	443.097273	+	+	+	+	+	+	+	+	+	+	+	+	+	+	+	+	+	+	+	+	+	+	+	+	+	+	+
Fisetinidol	275.091400	+	+	+	+	+	+	−	+	+	−	+	+	−	+	+	−	−	−	−	+	−	−	+	+	−	+	+
Guibourtinidol	259.096485	+	+	+	+	+	+	−	+	+	−	+	+	−	+	+	−	−	−	−	+	+	−	+	+	−	+	−
Oleanic acid	457.367622	+	+	+	+	+	+	+	−	+	+	+	+	+	−	+	+	−	+	+	−	+	+	+	+	+	−	+
Stigmasterol	413.377793	+	+	+	+	+	+	+	−	+	+	+	+	+	−	+	+	+	+	−	+	+	+	+	+	+	+	+
Resveratrol	229.085921	+	+	+	+	+	+	−	−	+	−	−	−	−	−	−	+	−	+	−	−	−	−	−	−	−	+	−
Myo-inositol	181.070665	−	+	+	+	+	+	−	+	+	−	+	−	−	+	−	−	+	+	−	+	+	−	+	−	−	−	−
Taxifolin	305.065579	+	+	+	+	+	+	+	+	+	+	+	+	+	+	+	+	+	+	+	+	+	+	+	+	+	+	+
Pelargonidin	271.060100	+	+	+	+	+	+	−	+	+	−	+	+	−	+	+	−	+	+	−	−	−	−	+	+	−	+	+
Malvidin	331.081229	−	−	−	−	−	−	−	+	−	−	+	+	−	+	+	+	+	+	−	+	+	−	+	+	−	+	+
Cyanidin-3-glucoside chloride	485.084516	+	+	+	+	+	+	−	−	+	−	−	−	−	−	−	−	−	−	−	−	−	−	−	−	−	−	−
Quinic acid	193.070665	+	+	+	+	+	+	−	+	+	−	+	+	−	+	+	−	+	+	−	+	+	−	+	+	−	+	+
Citric acid+Na	215.016223	+	−	−	−	−	+	−	+	+	−	+	−	−	−	−	−	−	−	−	−	+	−	+	−	−	−	−
Azelaic acid	189.112135	−	−	−	−	−	−	−	−	−	−	−	−	−	−	−	−	+	−	−	−	−	−	−	−	−	−	−
Asiatic acid	489.357451	+	+	+	+	+	+	+	+	+	+	+	+	+	+	+	−	+	+	+	+	+	+	+	+	+	+	+
Total number of compounds		33	34	35	35	35	36	17	34	36	18	36	33	18	32	33	18	23	27	15	26	28	18	36	33	18	34	31
Sample number		1	2	3	4	5	6	7	8	6	9	10	11	12	13	14	15	16	17	18	19	20	9	10	11	21	22	23

* MGL—mature green leaves; ** YGL—young green leaves; *** GL—green leaves; **** YL—yellow leaves; MS—magnetic stirring; UA—ultrasounds assisted; M—maceration; e—ethanol; w—water; m—hydroethanolic mixture; +/−—indicates the presence/ absence of the compounds in each studied extract sample.

## Data Availability

Not applicable.

## Supplementary Materials

The following supporting information can be downloaded at: https://www.mdpi.com/article/10.3390/molecules28217328/s1, Figure S1: Different extracts of *Juglans regia* L (color variation); Figure S2: Standard curve of gallic acid for total polyphenol content assay (*TPC*); Figure S3: Standard curve of Trolox for Trolox equivalent antioxidant capacity assay (*TEAC*). Figures S4_1–S4_23 show the FT-ICR-MS spectra of all samples. Table S1. Biological activities of the main compounds identified by the FT-ICR-MS method.
