# Supplementary material for: Superior Valorisation of Juglans regia L. Leaves of Different Maturity through the Isolation of Bioactive Compounds
Source: Molecules. 2023 Oct 29;28(21):7328. doi: 10.3390/molecules28217328 (PMC10648215; doi:10.3390/molecules28217328)
Supplement: Supplementary file 1 [file molecules-28-07328-s001.zip › molecules-2652828-supplementary.pdf]

FIGURES

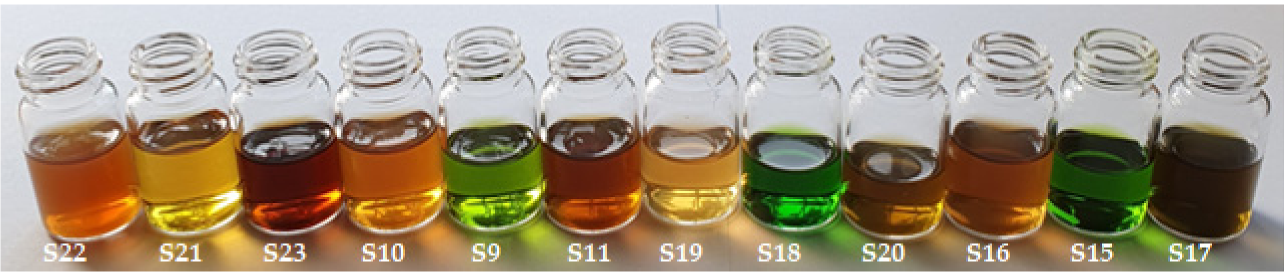

Figure S1 - Different extracts of *Juglans regia* L (color variation).

S22 - YL extraction with water; S21- YL extraction with ethanol; S23 - YL extraction with hydroethanolic mixture; S10 - MGL extraction with water; S9 - MGL extraction with ethanol; S11 - MGL extraction with hydroethanolic mixture; S19 - LG extraction with water; S19 - extraction with ethanol; S18 - extraction with hydroethanolic mixture; S16 - YGL extraction with water; S15 - YGL extraction with ethanol; S17 - YGL extraction with hydroethanolic mixture. All samples were subjected ultrasound assisted-extraction for 60 min

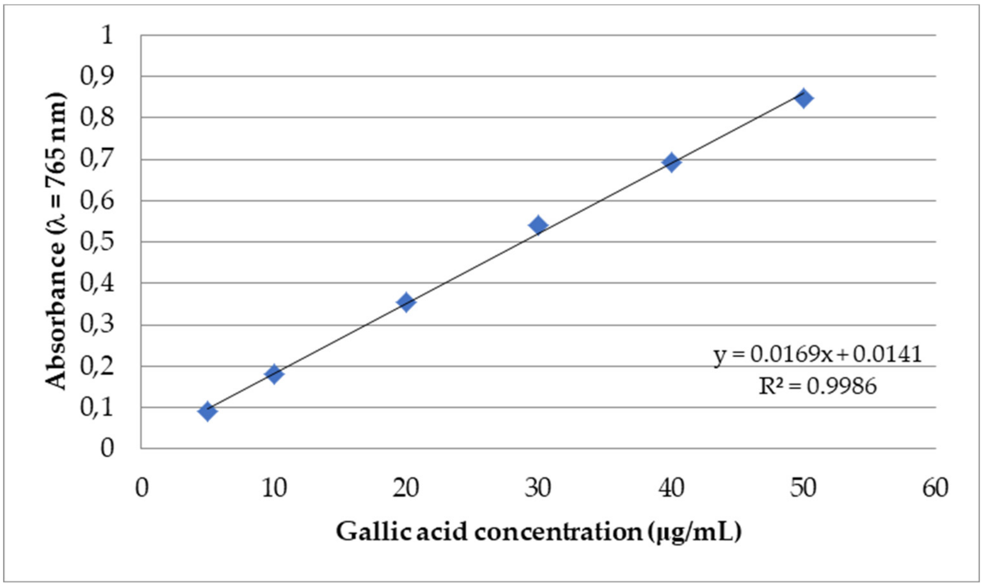

Figure S2 - Standard curve of gallic acid for total polyphenol content assay (TPC).

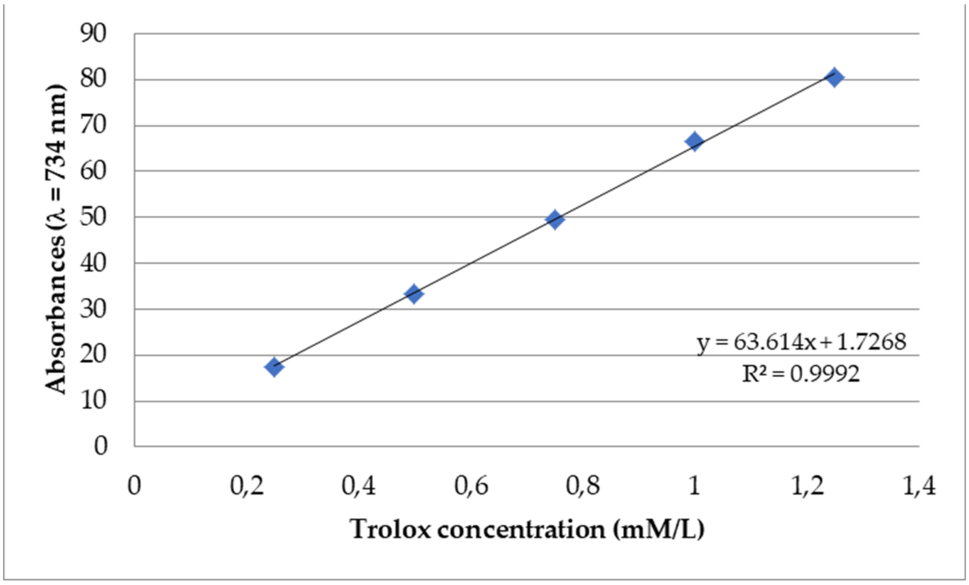

Figure S3 - Standard curve of Trolox for Trolox equivalent antioxidants capacity assay (TEAC).

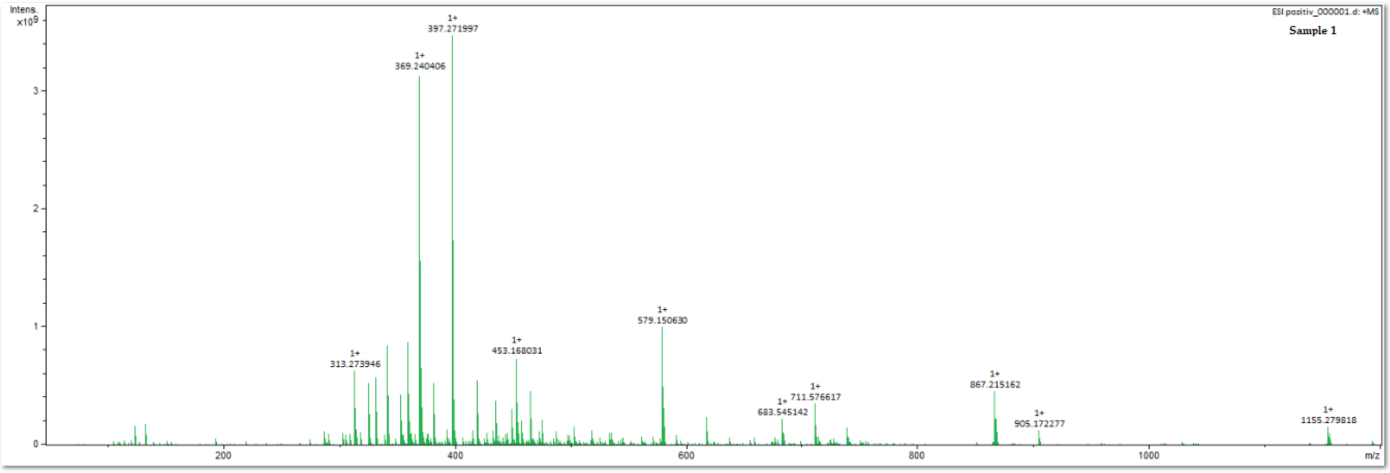

Figure S4_1 FT-ICR-MS spectrum of MGL - extraction by magnetic stirring with hydroethanolic mixture - 1 min.

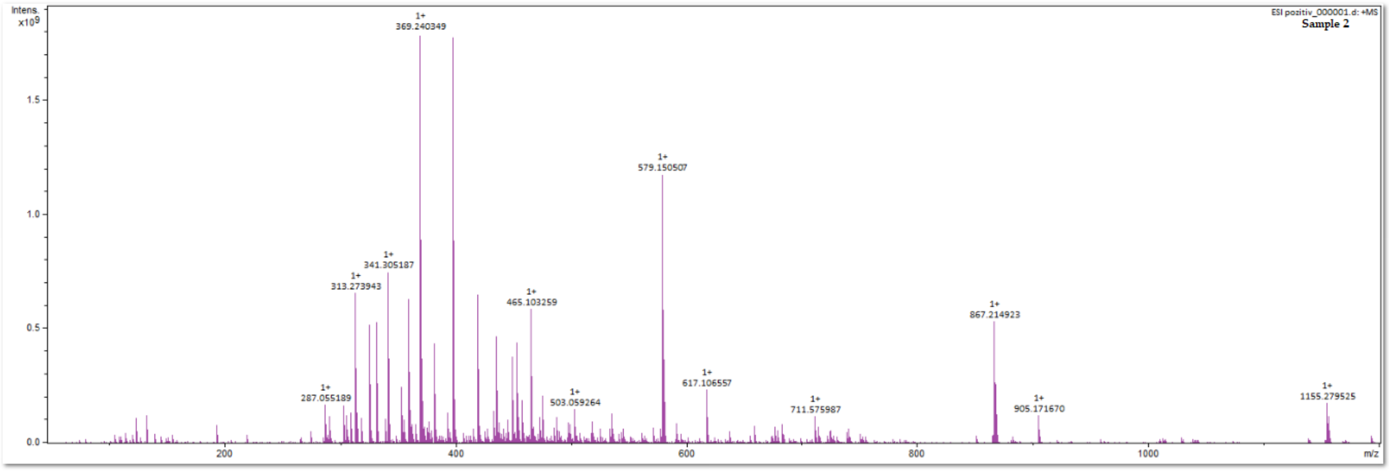

Figure S4_2 FT-ICR-MS spectrum of MGL - extraction by magnetic stirring with hydroethanolic mixture - 10 min.

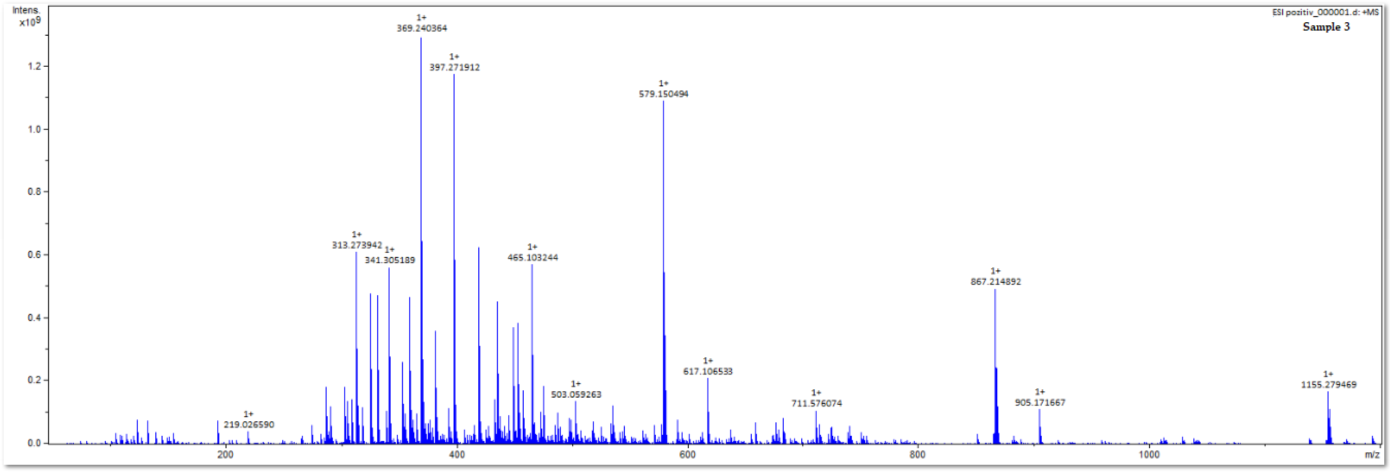

Figure S4_3 FT-ICR-MS spectrum of MGL - extraction by magnetic stirring with hydroethanolic mixture - 20 min.

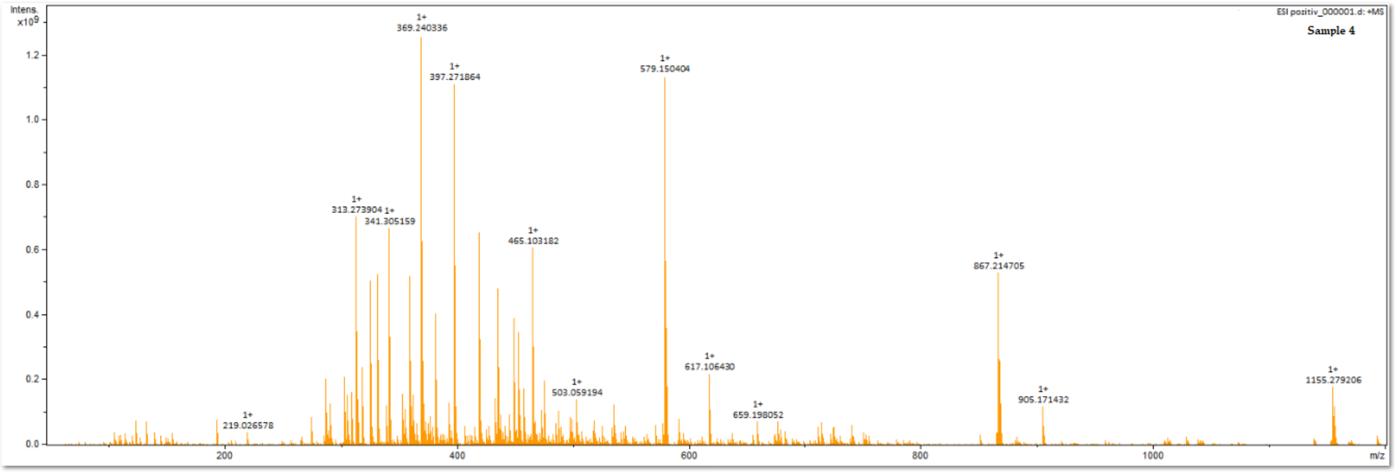

Figure S4_4 FT-ICR-MS spectrum of MGL - extraction by magnetic stirring with hydroethanolic mixture - 30 min.

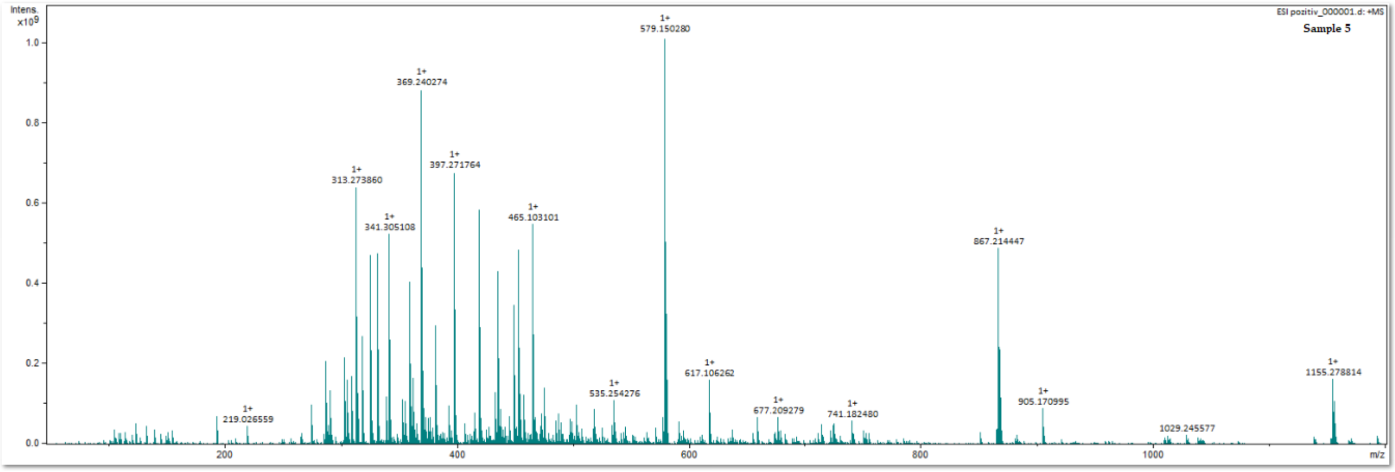

Figure S4_5 FT-ICR-MS spectrum of MGL - extraction by magnetic stirring with hydroethanolic mixture - 45 min.

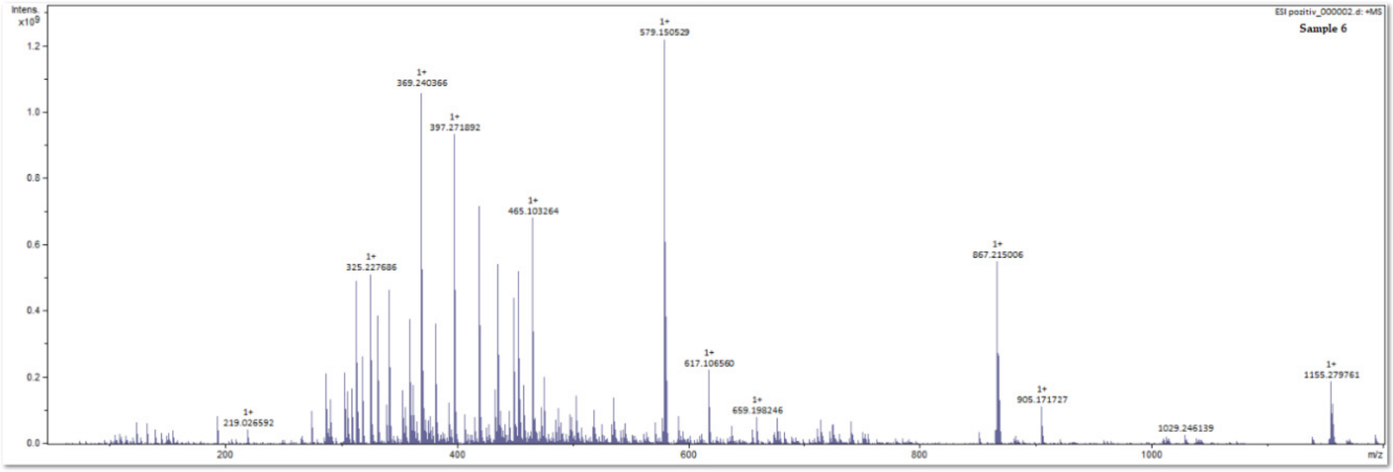

Figure S4_6 FT-ICR-MS spectrum of MGL - extraction by magnetic stirring with hydroethanolic mixture - 60 min.

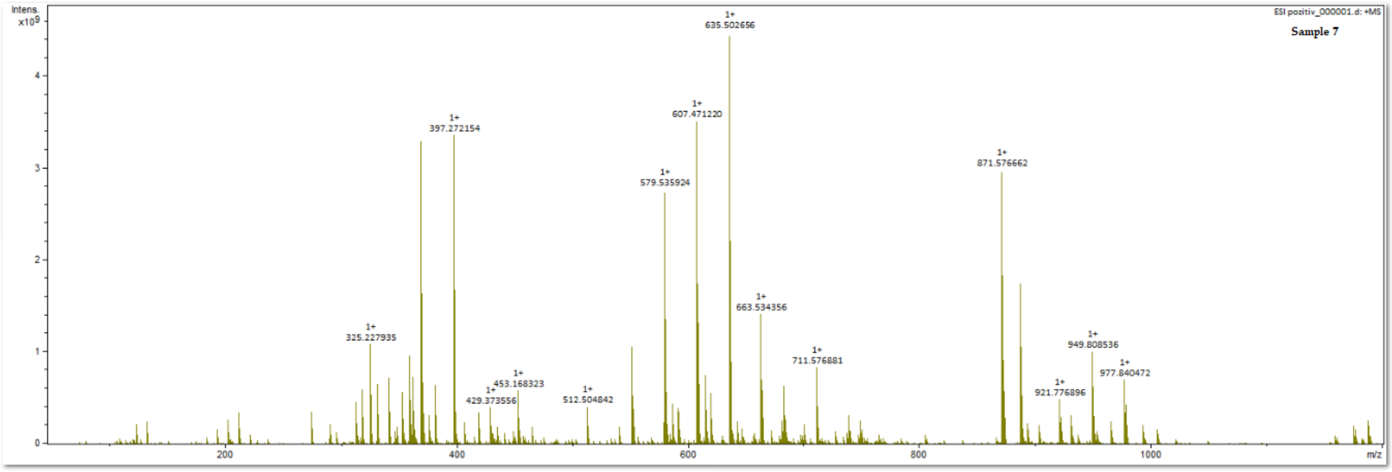

Figure S4_7 FT-ICR-MS spectrum of MGL - extraction by magnetic stirring with ethanol - 60 min.

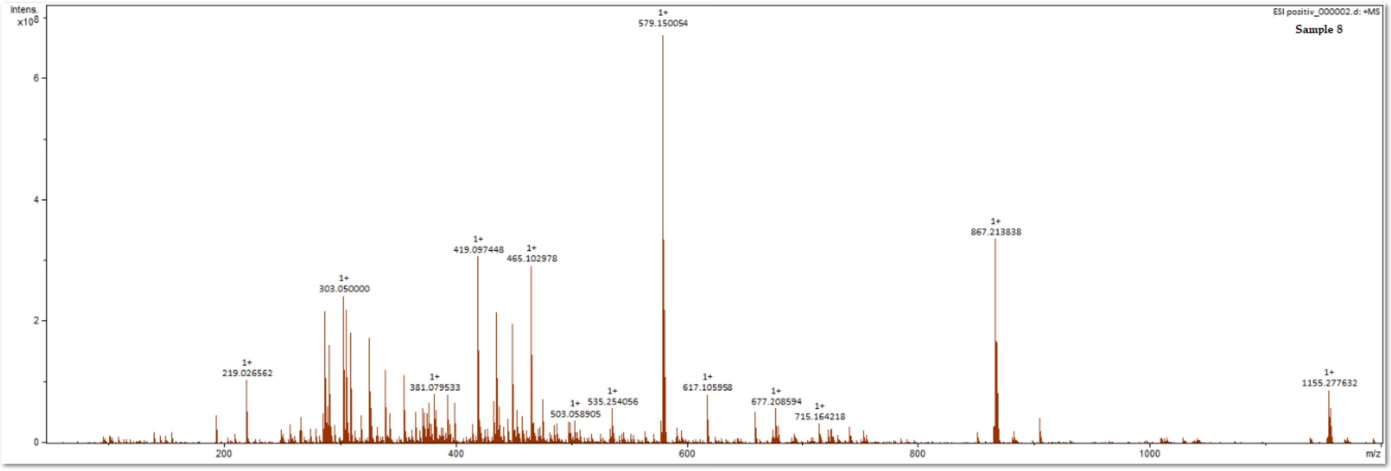

Figure S4_8 FT-ICR-MS spectrum of MGL - extraction by magnetic stirring with water - 60 min.

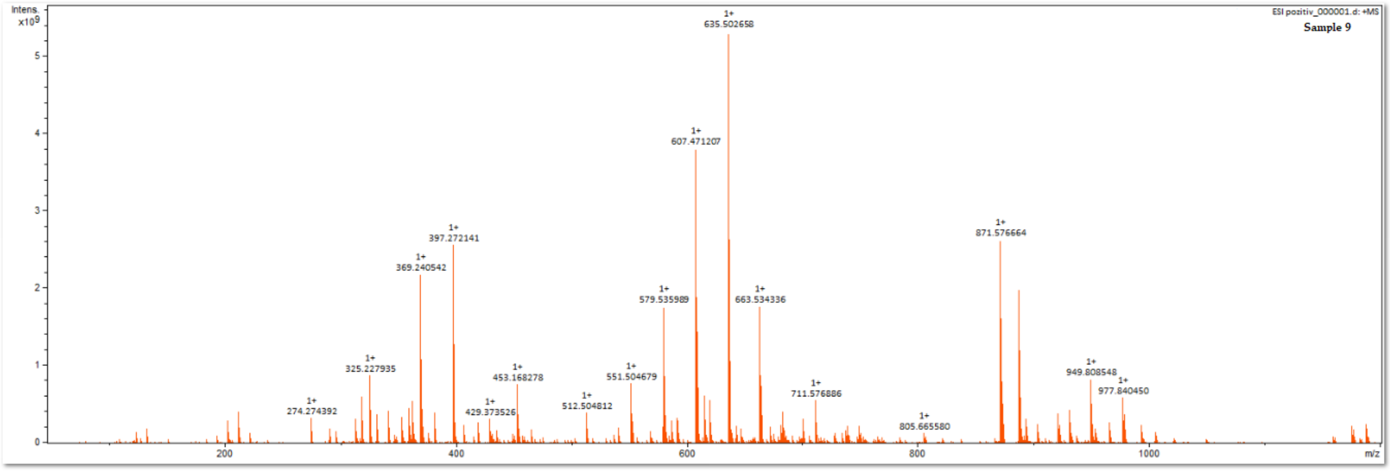

Figure S4_9 FT-ICR-MS spectrum of MGL - ultrasound assisted-extraction with ethanol - 60 min.

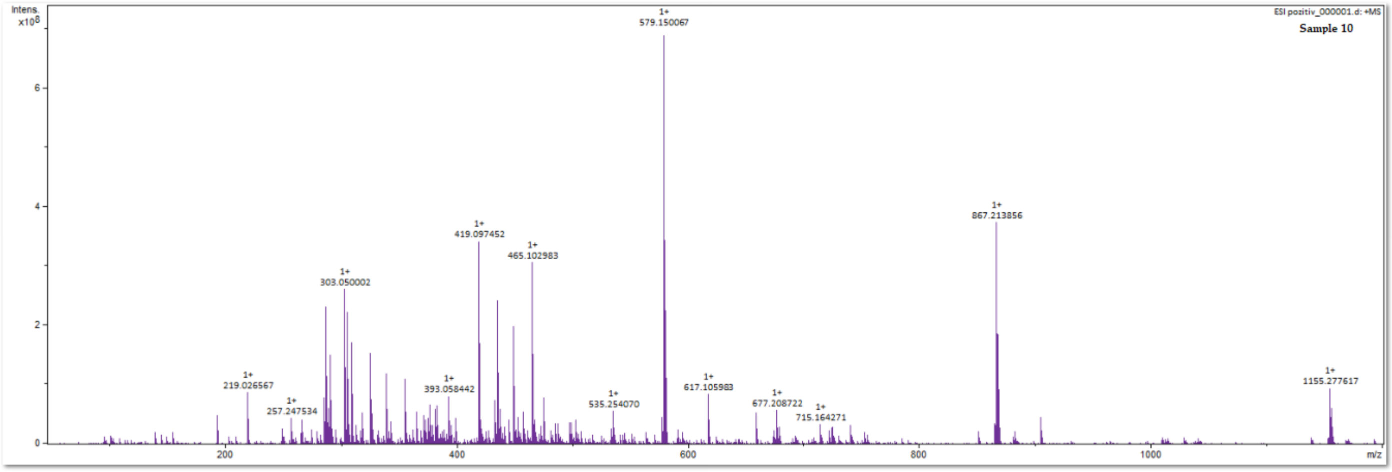

Figure S4_10 FT-ICR-MS spectrum of MGL - ultrasound assisted-extraction with water - 60 min.

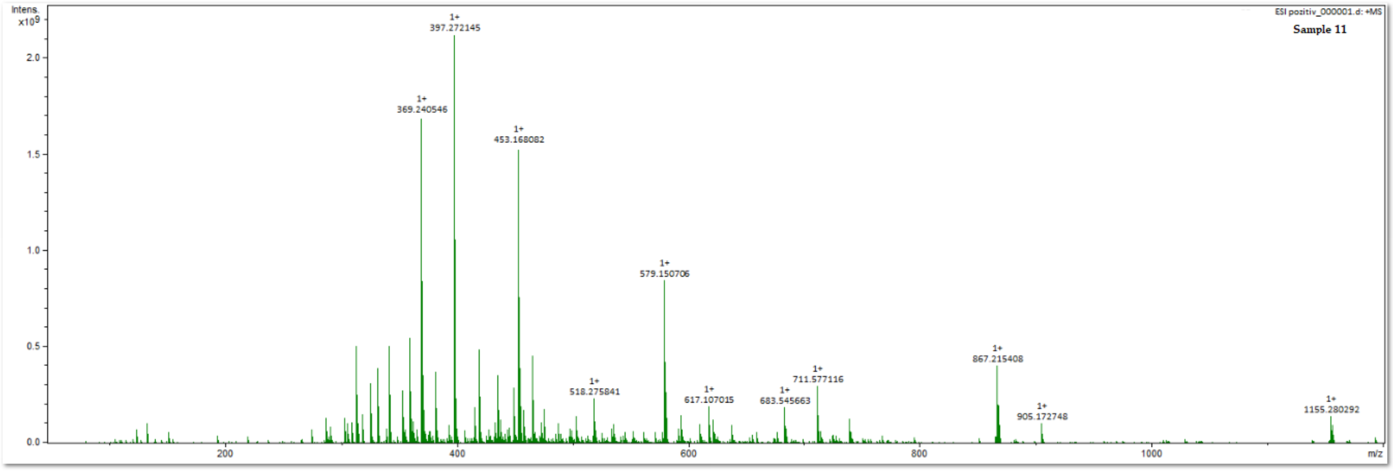

Figure S4_11 FT-ICR-MS spectrum of MGL - ultrasound assisted-extraction with hydroethanolic mixture - 60 min.

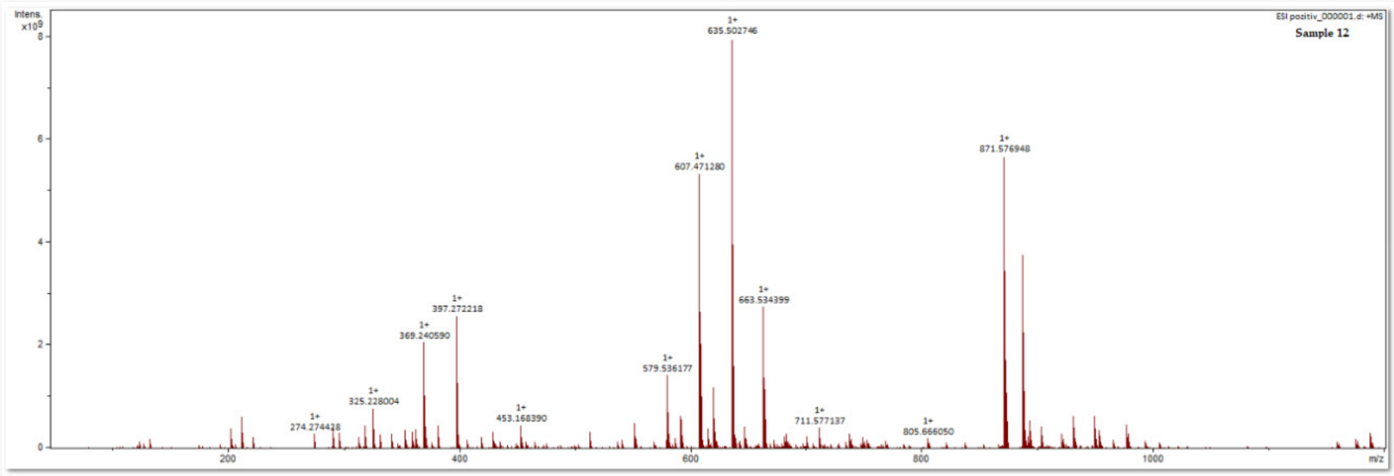

Figure S4_12 FT-ICR-MS spectrum of MGL - extraction by maceration with ethanol - 60 min.

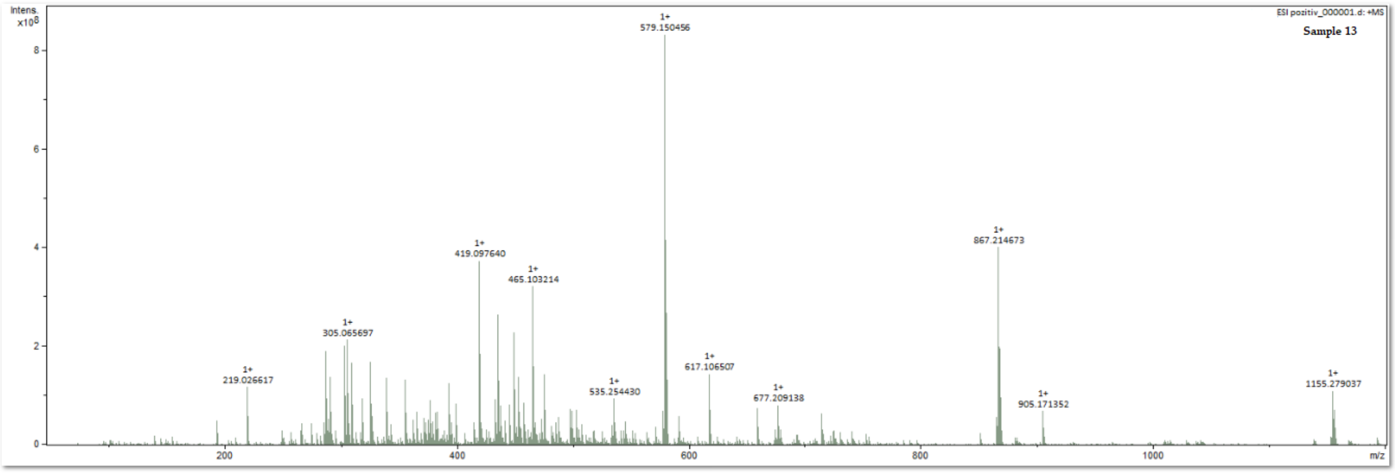

Figure S4_13 FT-ICR-MS spectrum of MGL - extraction by maceration with water - 60 min.

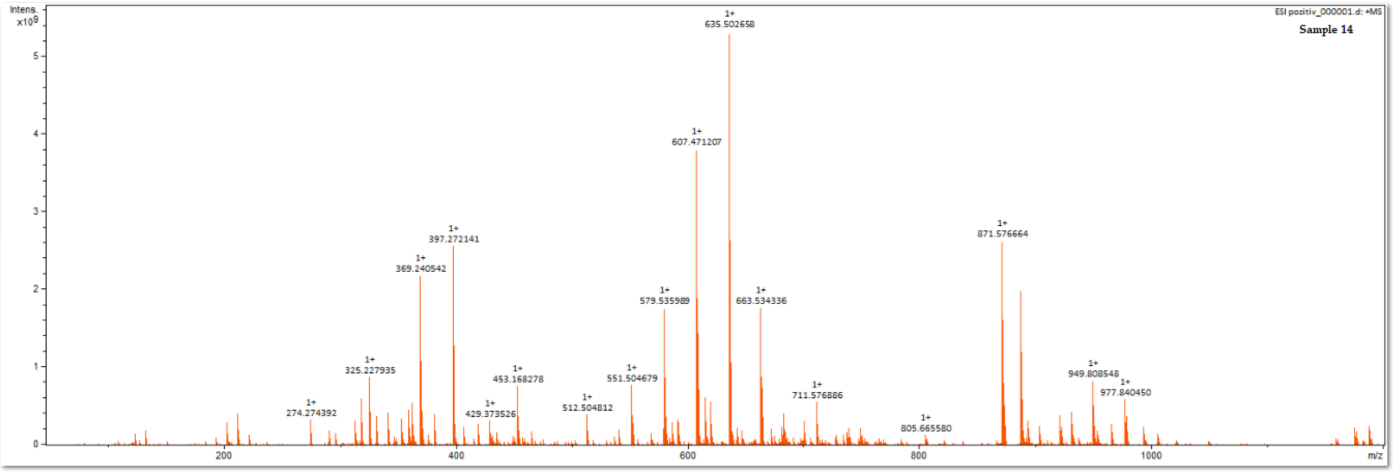

Figure S4_14 FT-ICR-MS spectrum of MGL - extraction by maceration with hydroethanolic mixture - 60 min.

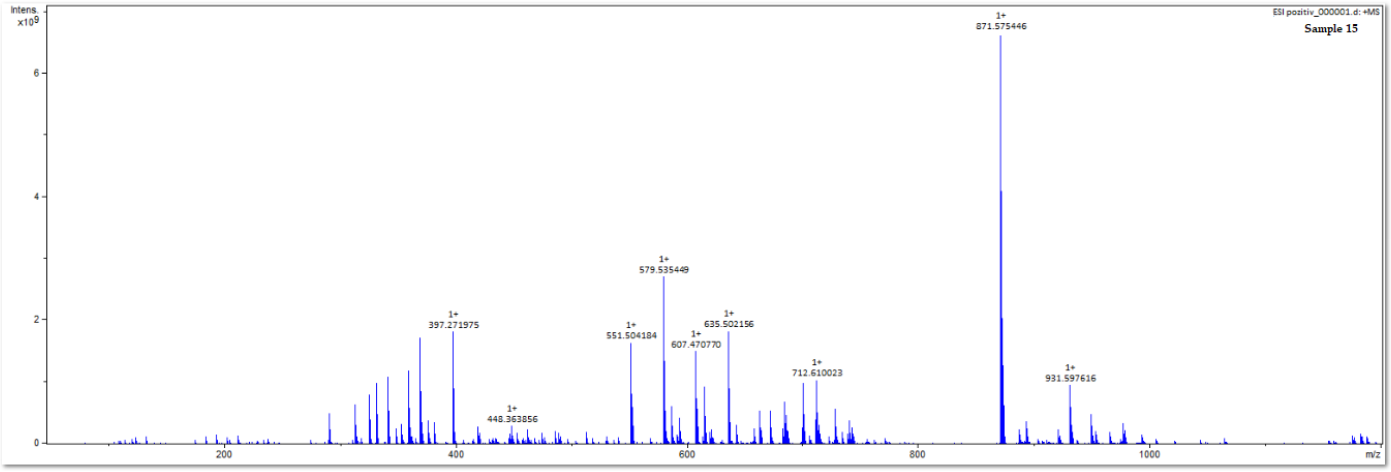

Figure S4_15 FT-ICR-MS spectrum of YGL - ultrasound assisted-extraction with ethanol - 60 min.

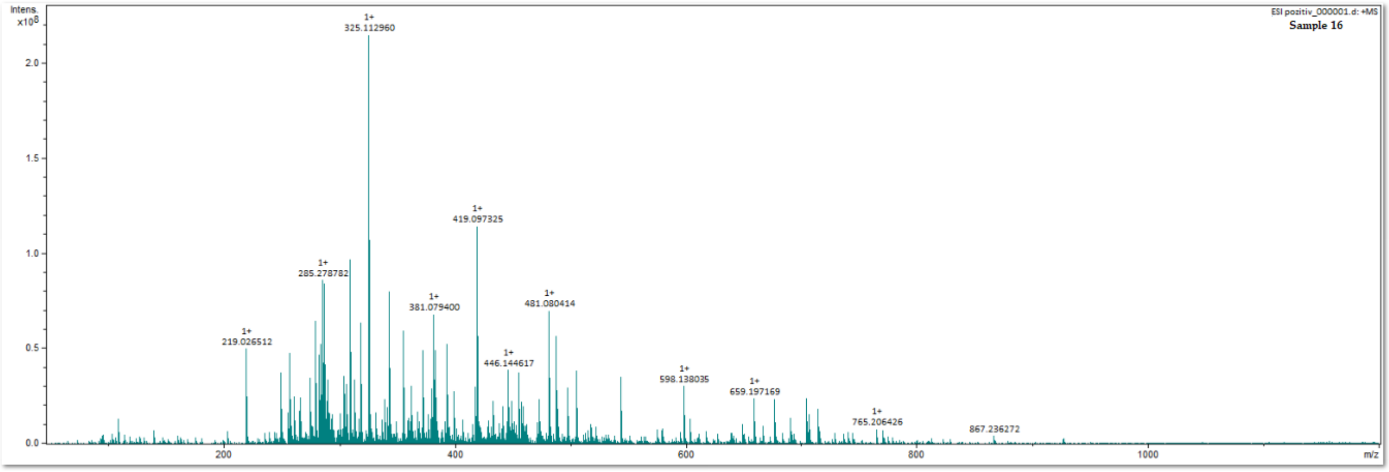

Figure S4_16 FT-ICR-MS spectrum of YGL - ultrasound assisted-extraction with water - 60 min.

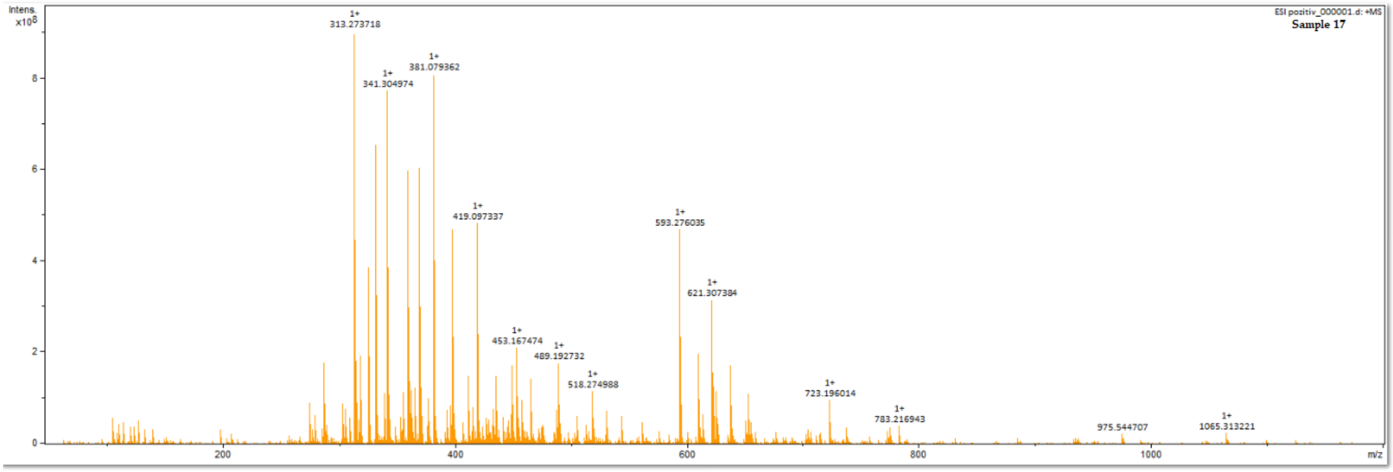

Figure S4_17 FT-ICR-MS spectrum of YGL - ultrasound assisted-extraction with hydroethanolic mixture - 60 min.

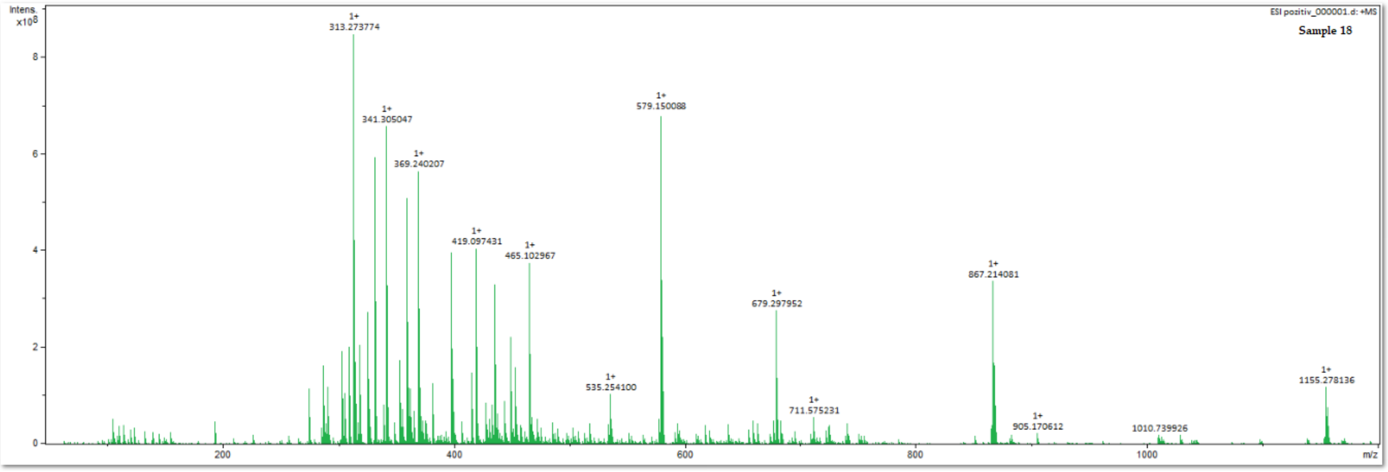

Figure S4_18 FT-ICR-MS spectrum of GL - ultrasound assisted-extraction with ethanol - 60 min.

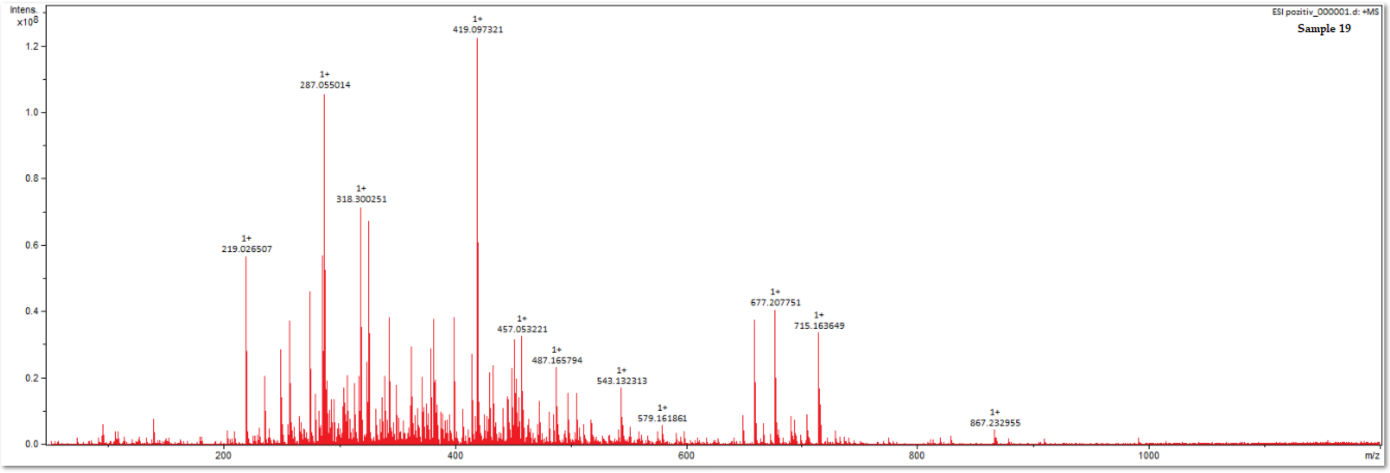

Figure S4_19 FT-ICR-MS spectrum of GL - ultrasound assisted-extraction with water - 60 min.

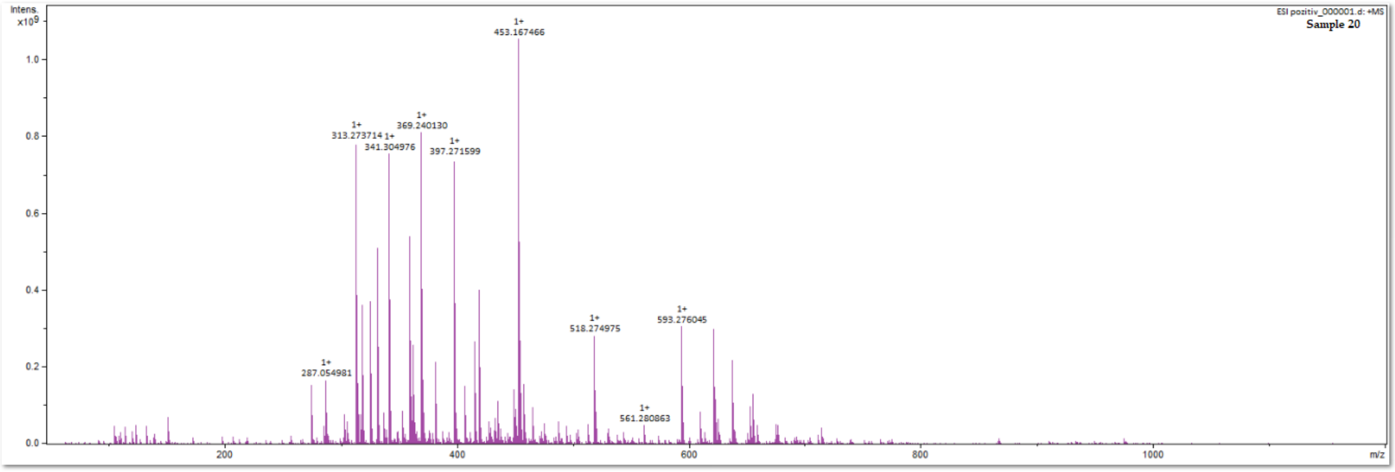

Figure S4_20 FT-ICR-MS spectrum of GL - ultrasound assisted-extraction with hydroethanolic mixture - 60 min.

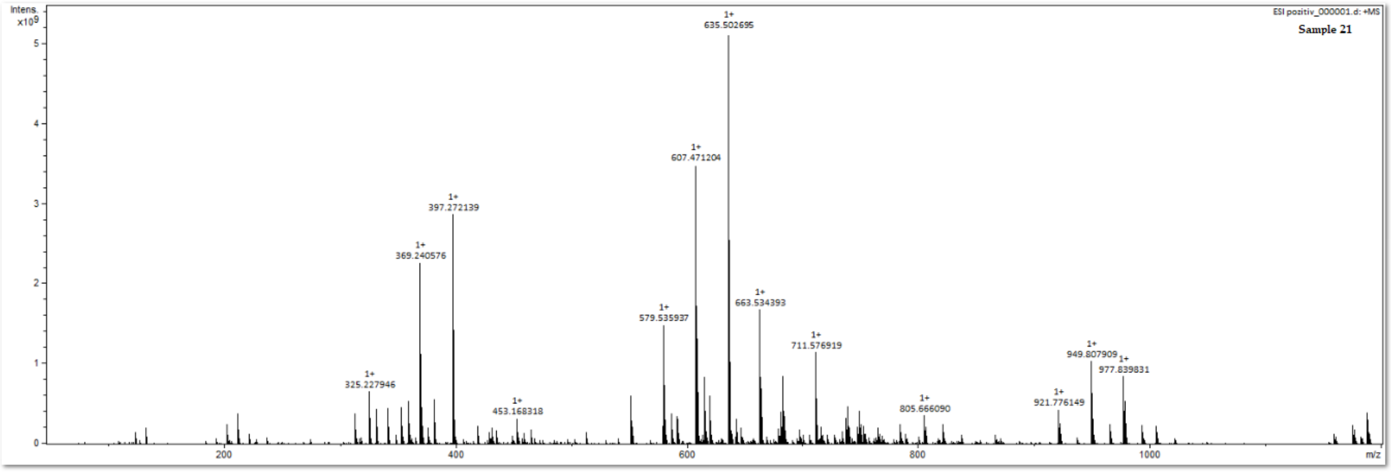

Figure S4_21 FT-ICR-MS spectrum of YL - ultrasound assisted- extraction with ethanol - 60 min.

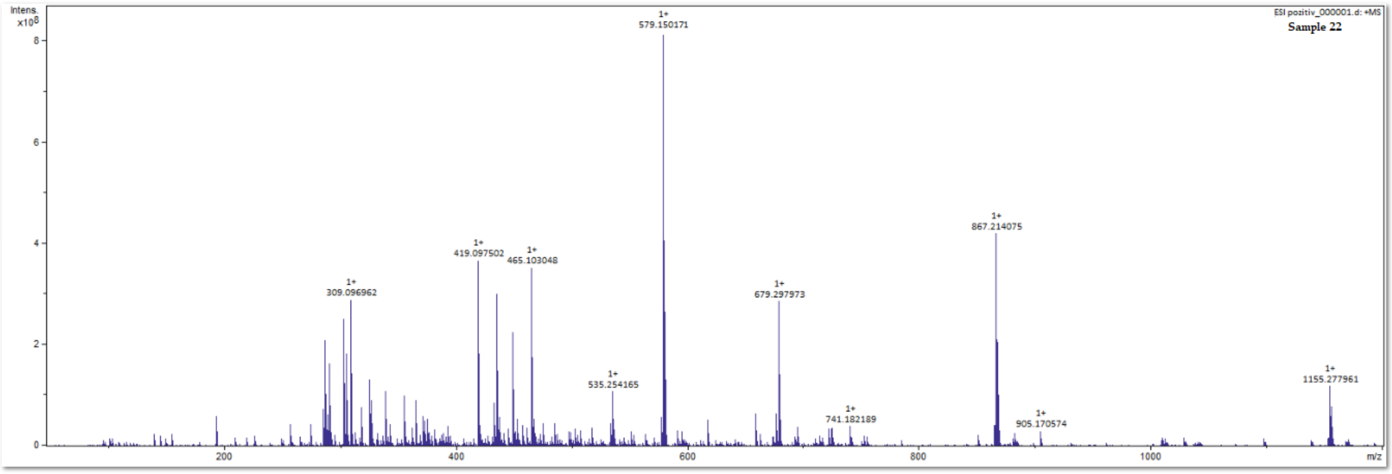

Figure S4_22 FT-ICR-MS spectrum of YL - ultrasound assisted-extraction with water - 60 min.

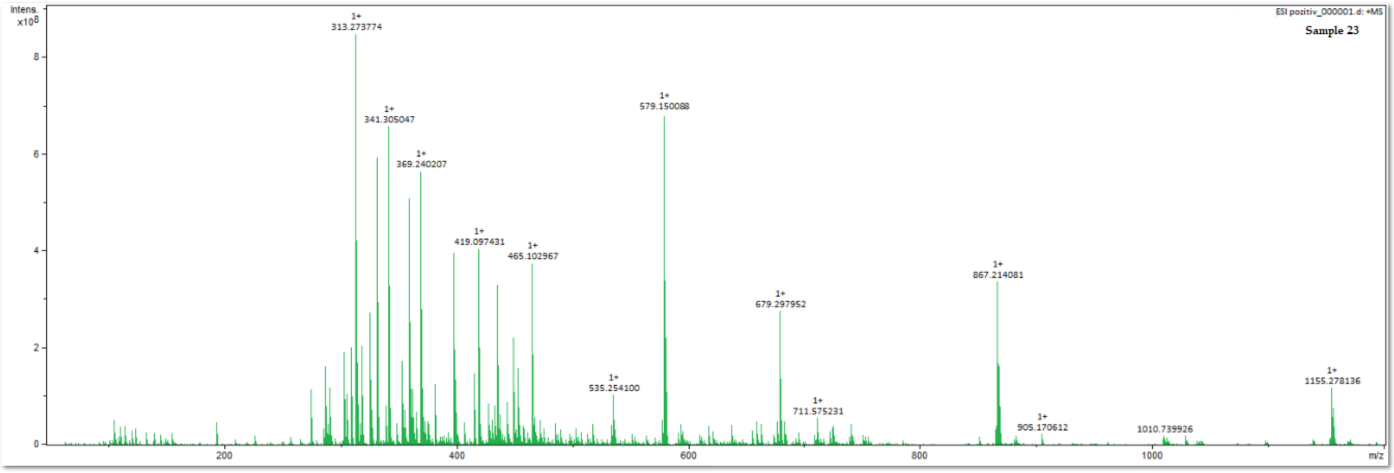

Figure S4_23 FT-ICR-MS spectrum of YL - ultrasound assisted-extraction with hydroethanolic mixture - 60 min.

TABLES

Table S1. Biological activities of the main compounds identified in the walnut leaf's extracts by means of FT-ICR-MS method.

Compound Class	Compound	Chemical formula	Biological activity [6,34,39,47-60]
	Caffeic acid	$C_9H_8O_4$	Antioxidant, anti-inflammatory and anticarginogenic activity
	Caftaric acid	$C_{13}H_{12}O_9$	Antioxidant, antidiabetic, anti-inflammatory, anticarcinogenic, antiobesity
	Chlorogenic acid	$C_{16}H_{18}O_9$	Antioxidant, lower blood pressure
	Ellagic acid	$C_{14}H_6O_8$	Cytotoxic, anti-proliferative
	Ferulic acid	$C_{10}H_{10}O_4$	Anticancerous
Phenolic	Juglone	$C_{10}H_6O_3$	Antibacterial, antiviral, anticancer, antioxidant
	p-coumaric acid	$C_9H_8O_3$	Antioxidant
	Sinapate	$C_{11}H_{12}O_5$	Antioxidant, antimicrobial
	3-p-coumaroylquinic acid	$C_{16}H_{18}O_8$	Antibacterial
	Quercetin 3-o-arabinoside	$C_{20}H_{18}O_{11}$	Antioxidant, anti-inflammatory
	Quercetin 3-rhamnoside	$C_{21}H_{20}O_{11}$	Antioxidant, anti-inflammatory, anti-cancer and lipid peroxidation inhibitory activities
	Quercetin 3-o-pentoside	$C_{25}H_{26}O_{15}$	Antioxidant
	Quercetin	$C_{15}H_{10}O_7$	Analgesic, antibacterial, antiviral
	Myricetin	$C_{15}H_{10}O_8$	Anticancer, antidiabetic, antibacterial, antiviral
	Kaempherol	$C_{15}H_{10}O_6$	Acute and chronic inflammation
	Kaempferol 3-o-arabinoside	$C_{20}H_{18}O_{10}$	Antioxidant
Flavonoid	Kaempferol-3-o-rhamnoside	$C_{21}H_{20}O_{10}$	Antioxidant, anti-tumor
	Isokaempferide	$C_{16}H_{12}O_6$	Hepatoprotective, antimicrobial, anti-proliferative effect
	Catechin hydrate	$C_{15}H_{14}O_6+H_2O$	Antioxidant, anticancer
	Hyperoside	$C_{21}H_{20}O_{12}$	Anti-inflammatory, anti-oxidant stress, anti-swelling, anti-bacterial and anti-viral effects
Flavone	Luteolin	$C_{15}H_{10}O_6$	Anticancer, anti-inflammatory
Flavanone	Naringenin	$C_{15}H_{12}O_5$	Antiviral, antibacterial, antioxidant

Flavonone	Hesperetin	$C_{21}H_{20}O_{12}$	Scavenging activity and cardioprotective activity
Flavanol	Catechin	$C_{15}H_{14}O_6$	Antioxidant, anti-inflammatory
	Epigallocatechin	$C_{15}H_{14}O_7$	Antioxidant, antitumor
	Epigallocatechin gallate	$C_{22}H_{18}O_{11}$	Reduce inflammation, aid weight loss, help prevent heart and brain disease
	Fisetinidol	$C_{15}H_{14}O_5$	Antioxidant, antibacterial
	Guibourtinidol	$C_{15}H_{14}O_4$	Antioxidant, prevent HIV-1
Tereponoid	Oleanic acid	$C_{30}H_{48}O_3$	Dyslipidemia, diabetes, and metabolic syndrome
Steroid	Stigmasterol	$C_{29}H_{48}O$	Anticancerous, dyslipidemia, diabetes, and metabolic syndrome
Vitamin	Resveratrol	$C_{14}H_{12}O_3$	Anti-inflammatory, anti-oxidant and anti-carcinogenic effects
	Myo-inositol	$C_6H_{12}O_6$	Prevent and improve insulin resistance
	Taxifolin	$C_{15}H_{12}O_7$	Antioxidant, support immune, respiratory, skin and cellular health benefits
Anthocyanidin	Pelargonidin	$C_{15}H_{11}O_5+$	Antioxidant
	Malvidin	$C_{17}H_{15}O_7+$	Antioxidant, anti-inflammatory
	Cyanidin 3-glucoside chloride	$C_{21}H_{21}ClO_{11}$	Antioxidant, anti-inflammatory, anti-ischemic, anti-cancer
Other compounds	Quinic acid	$C_7H_{12}O_6$	Radioprotection, anti-neuroinflammatory, antioxidant
	Citric acid	$C_6H_8O_7$	Antibacterial, antioxidant, anti-inflammatory
	Azelaic acid	$C_9H_{16}O_4$	Anti-inflammatory, antioxidant
	Asiatic acid	$C_{30}H_{48}O_5$	Antioxidant, anti-aging
